# Genome-wide analysis of regulatory G-quadruplexes affecting gene expression in human cytomegalovirus

**DOI:** 10.1371/journal.ppat.1007334

**Published:** 2018-09-28

**Authors:** Subramaniyam Ravichandran, Young-Eui Kim, Varun Bansal, Ambarnil Ghosh, Jeonghwan Hur, Vinod Kumar Subramani, Subhra Pradhan, Myoung Kyu Lee, Kyeong Kyu Kim, Jin-Hyun Ahn

**Affiliations:** Department of Molecular Cell Biology, Sungkyunkwan University School of Medicine, Samsung Medical Center, Suwon, Republic of Korea; Wistar Institute, UNITED STATES

## Abstract

G-quadruplex (G4), formed by repetitive guanosine-rich sequences, is known to play various key regulatory roles in cells. Herpesviruses containing a large double-stranded DNA genome show relatively higher density of G4-forming sequences in their genomes compared to human and mouse. However, it remains poorly understood whether all of these sequences form G4 and how they play a role in the virus life cycle. In this study, we performed genome-wide analyses of G4s present in the putative promoter or gene regulatory regions of a 235-kb human cytomegalovirus (HCMV) genome and investigated their roles in viral gene expression. We evaluated 36 putative G4-forming sequences associated with 20 genes for their ability to form G4 and for the stability of G4s in the presence or absence of G4-stabilizing ligands, by circular dichroism and melting temperature analyses. Most identified sequences formed a stable G4; 28 sequences formed parallel G4s, one formed an antiparallel G4, and four showed mixed conformations. However, when we assessed the effect of G4 on viral promoters by cloning the 20 putative viral promoter regions containing 36 G4-forming sequences into the luciferase reporter and monitoring the expression of luciferase reporter gene in the presence of G4-stabilizing chemicals, we found that only 9 genes were affected by G4 formation. These results revealed promoter context-dependent gene suppression by G4 formation. Mutational analysis of two potential regulatory G4s also demonstrated gene suppression by the sequence-specific G4 formation. Furthermore, the analysis of a mutant virus incapable of G4 formation in the UL35 promoter confirmed promoter regulation by G4 in the context of virus infection. Our analyses provide a platform for assessing G4 functions at the genomic level and demonstrate the properties of the HCMV G4s and their regulatory roles in viral gene expression.

## Author summary

Although a number of G4-forming sequences are predicted in the herpesviral genomes and some G4s are characterized individually, why so many G4s are present in the genome and whether they are all functional are not yet clear. Our genome-wide bioinformatic sequence analyses and biophysical stability and structure assays of G4s present in the HCMV genome revealed that most G4s present in the HCMV gene regulatory regions form a stable G4 structure. However, it was found that only a few HCMV G4s suppress viral gene expression when the effect of G4 on gene expression was examined by *in vivo* reporter assay. Therefore, this study demonstrates that G4 activity relies on the promoter context, providing a new insight into understanding gene regulation by G4 structures. This study also provides evidence that G4 plays a regulatory role in gene expression during HCMV infection.

## Introduction

Repetitive guanosine-rich (G-rich) sequences connected by short stretches of nucleotides in the genome of an organism can fold into a distinct type of tertiary structure known as a G-quadruplex (G4). Four guanine bases connected with each other through Hoogsteen hydrogen bonding form a square planar structure known as a guanine tetrad or G-tetrad. Multiple G-tetrads can stack on top of each other in a G4 structure, which can be further stabilized in the presence of monovalent or divalent cations [1–3].

Since the presence of G4s in the human genome was first observed in the telomere region and their structure was proposed [4–6], many studies have confirmed their existence in other parts of the genome such as the promoter [7], the 5′ and 3′ untranslated regions (UTRs) [8–10], and even the coding region [11, 12]. Regarding the functional aspect, G4 can cause hindrance to replication, recombination, and transcription depending on its position in the genome [13]. Furthermore, the translational machinery is affected by the formation of G4 structure in RNA, suggesting that G4 has diverse regulatory roles at both DNA and RNA levels [2, 13]. G4 formation and functions in cells can be greatly influenced by proteins that can stabilize or resolve G4 structures [14, 15]. In addition, G4 stability can also be enhanced by several ligands that specifically recognize and bind G4 structures [2, 16]. In this regard, G4-stabilizing ligands have been extensively studied for therapeutic purposes [17, 18], mostly targeting G4s present in the promoters of oncogenes such as C-MYC, K-RAS, and BCL2 [7, 19–22]. G4-binding ligands have also been studied for the treatment of neurodegenerative diseases such as amyotrophic lateral sclerosis (ALS), motor neuron disease (MND), and frontotemporal dementia (FTD) [23].

Bioinformatics prediction based on G-rich sequences reveals that a number of putative G4-forming sequences are present in the genomes of almost all species belonging to three domains, bacteria, archaea, and eukaryota [24–29], although their number varies. For example, the number of G4-forming sequences in the human genome is predicted to be approximately 376,000 [12], while those in *Escherichia coli* are 6,754 [27]. Considering these numbers, the human genome contains an average of 0.12 putative G4 motifs per kb, whereas *E*.*coli* contains an average of 1.45 G4 motifs per kb. Recent high-throughput sequencing analyses identified more than 700,000 G4s in the human genome [30]. Nevertheless, why so many G4s are present in the genome and whether they are all functional are yet unclear. Most studies on the G4 function have been done on individual G4s. However, a genome-wide functional analysis is required for answering those questions and understanding the biological significance of G4s.

G4s have also been reported in diverse RNA and DNA viruses. In RNA viruses, such as retroviruses, flaviviruses, and filoviruses, G4s present in the long terminal repeat (LTR), in the UTR, or in the coding region modulate gene expression and recombination [31–38]. In DNA viruses, G4s present in the genomes of adeno-associated virus and human herpesviruses regulate viral DNA replication [39–43], while G4s in the promoter region of hepatitis B virus (HBV) and in the mRNA of Epstein-Barr virus modulate transcription and translation [44] [45, 46]. However, most of these studies aimed to understand the role of individual viral G4s, while genome-wide studies using the entire viral genomes are limited. Notably, a recent genome-wide bioinformatics study demonstrated that relatively higher density of G4-forming sequences was found in herpesvirus genomes compared to that in human and mouse genomes [47].

Human cytomegalovirus (HCMV), also known as human herpesvirus-5 (HHV-5), is a member of the β-herpesvirus subfamily and contains a 235-kb double-stranded DNA genome. HCMV infection is usually asymptomatic in healthy individuals, but often harmful or life-threatening for newborns and immune-compromised individuals [48]. A recent bioinformatics study has proposed the presence of a high number of G4-forming sequences in the HCMV genome [47]. Although G4s have been shown to play a key role in the regulation of the virulence genes of the virus [49, 50], the roles of the HCMV G4s during infection have not been studied at the genomic level.

In this study, we analyzed the G4s present in the putative promoter or regulatory regions of genes in the HCMV genome to understand their roles in gene expression. Using bioinformatics analysis, we identified 36 putative G4-forming sequences and investigated their ability to form stable G4 structures by circular dichroism (CD) spectroscopy and melting temperature (Tm) analyses. By transfecting the reporter constructs, in which the reporter gene was driven by the G4-containing viral promoter, into primary human foreskin fibroblasts (HF) in the presence or absence of G4-stabilizing ligands, we evaluated the effect of G4 formation on the regulation of viral gene expression. Finally, we examined the influence of G4 formation on the expression of key regulatory HCMV genes during virus infection using mutagenesis approaches.

## Results

### Identification of putative G4-forming sequences in the promoter regions of HCMV

We first applied bioinformatics analysis to search for putative G4-forming sequences in the HCMV genome using the “G(3–6)N(1–7)G(3–6)N(1–7)G(3–6)N(1–7)G(3–6)” schema. Through this analysis, we identified 35 putative G4-forming sequences classified as conventional G4s that conformed to this schema. However, since it was revealed that many nonconventional G4s (bulged and long-loop) were also found by recent high-throughput sequencing results in human genome [30], we further explored G4s with long-loop and bulged signatures in the HCMV genome by adjusting the new search schema accordingly. We found 263 putative G4-forming sequences—39 conventional, 75 long-loop, and 149 bulged (S1 Appendix), and thus the G4 frequency is 1.11 G4 motifs per kb. These included overlapping G4s extracted from extremely long sequences containing contiguous G-tracts, which were broken down into individual sequences to explore probable G4-folding topologies. Compared to the human genome, in which 43% G4s were unconventional [30], HCMV contained a relatively large portion of unconventional G4s (80%).

**Fig 1:**
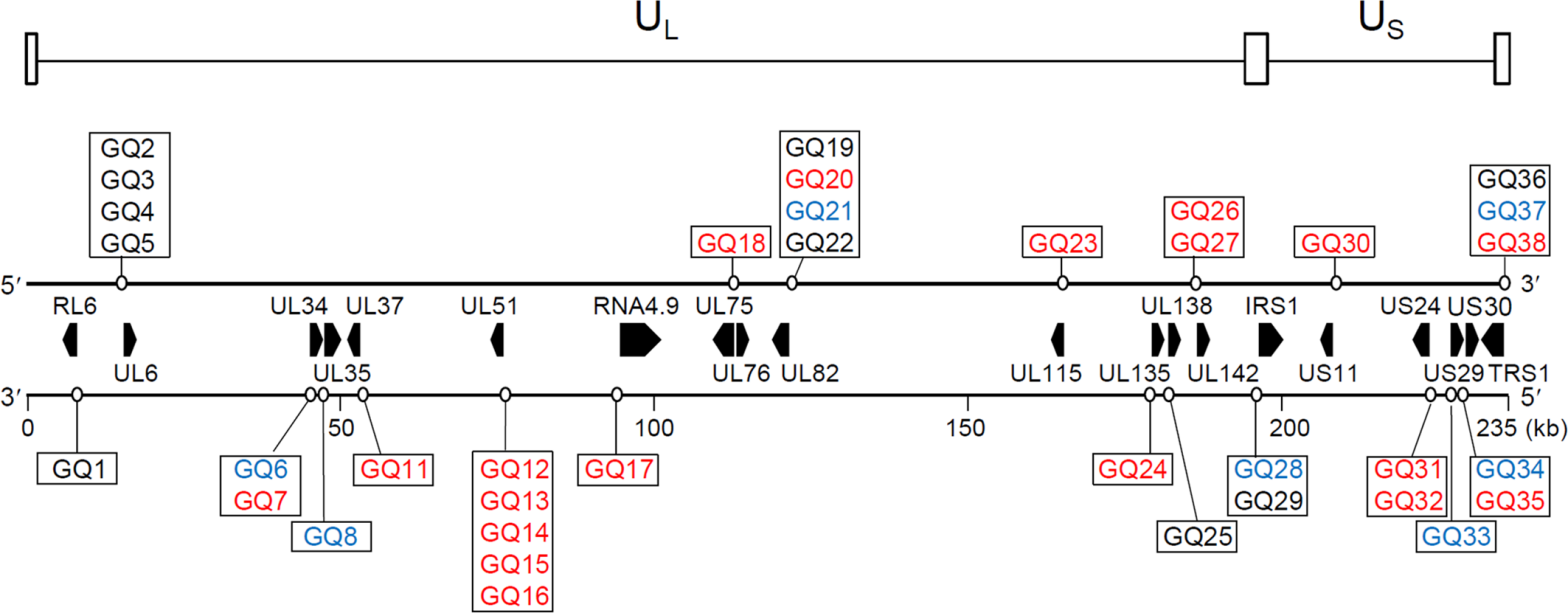
Schematic representation of the positions of genes and predicted 36 GQs associated with the putative promoter or regulatory regions in the HCMV genome. Among 38 GQs (GQ1~GQ38), which were initially annotated between -500 and +100 with respect to the translation initiation sites of genes (S1 Table), 36 GQs that were analyzed in this study are presented in the diagram. The x-axis represents the genomic scale or base positions. Two strands of HCMV DNA are separately shown as parallel lines. Approximate locations and strand positions of GQs are shown with open circles. Location and orientation of the associated genes are indicated as black bar pointers between the two strands. The types of G4s are differentiated by different font colors: type-I (conventional), blue; type-II (long-loop), black; and type-III (bulged), red. The unique long (U_L_) and unique short (U_S_) regions of the HCMV genome and the terminal and internal repeat regions (open boxes) are indicated at the top of the figure.

In the HCMV genome, the transcription start sites and the TATA boxes have been identified for only a limited number of the genes. Therefore, to identify the regulatory GQs affecting viral gene expression, we focused on 38 putative G4-forming sequences (denoted as GQ1 to GQ38) between -500 and +100 with respect to the translation initiation sites for 172 HCMV genes (S1 Table). Among them, 5 G4-forming sequences overlapped with the ATG start codon (GQ2, GQ3, GQ4, GQ5, and GQ19), and 2 sequences (GQ9 and GQ10) were located far downstream from the ATG codon (S2 Table). Since we aimed to explore the role of G4 in the putative promoter regions, we excluded GQ9 and GQ10 from further studies. Therefore, a total of 36 putative G4-forming sequences, which were associated with 20 genes, were analyzed for their G4 formation, stability, and effect on the promoter activity (Fig 1). GQ18 was associated with both UL75 and UL76. In addition, 5 genes (UL34, UL82, IRS1, US30, and TRS1) harbored more than one type of putative G4-forming sequence. GQ29 and GQ36, which were found upstream of IRS1 and TRS1, respectively, showed the same G4 sequence. This was also found in the case of GQ28 and GQ37. Among 36 GQs analyzed, 7 GQs showed a conventional signature (Type I), 10 belonged to long-loop type (Type II), and 19 showed the bulged type (Type III) (Fig 1; S1 Table).

### Biophysical analysis of HCMV G4s

**Fig 2:**
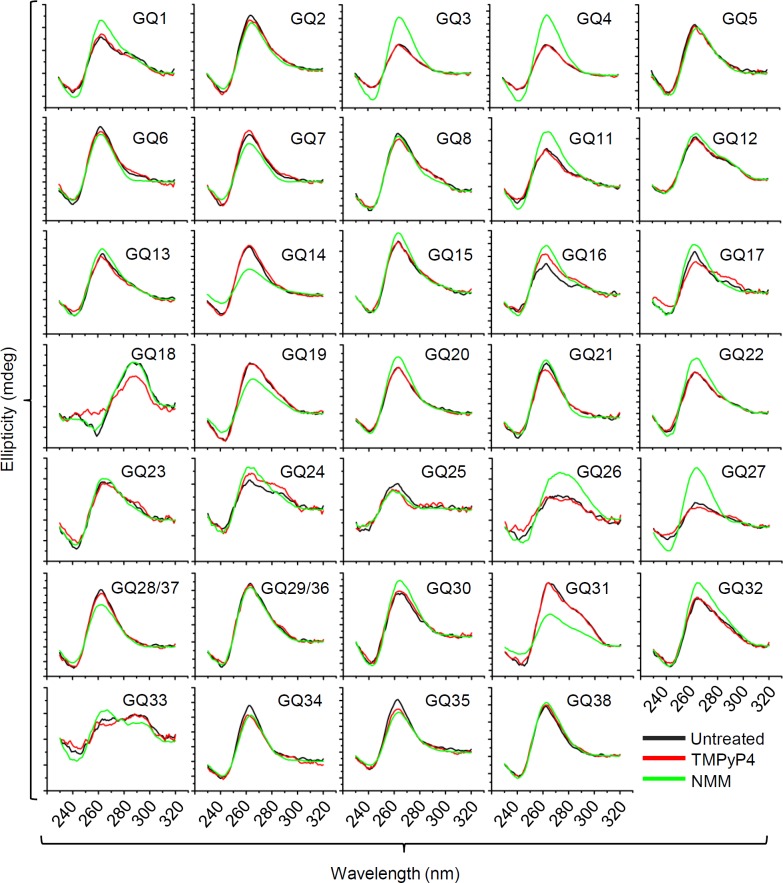
CD spectra of HCMV G4 oligos in the absence and presence of G4 stabilizers TMPyP4 and NMM. Comparison of CD spectra of 36 G4-oligonucleotides in the presence of G4 ligands, TMPyP4 and NMM. Fifteen μM DNA oligos were annealed in the presence of 10 mM Tris-HCl [pH 7.5] and 100 mM KCl buffer with or without 30 μM TMPyP4 and NMM (DNA to chemical ratio 1:2). Each spectrum was an average of 3 accumulations in the wavelength range between 230–320 nm. The spectra were blanked with buffer only. Note that the sequences of GQ28 and GQ37 were identical, as GQ29 and GQ36.

**Fig 3:**
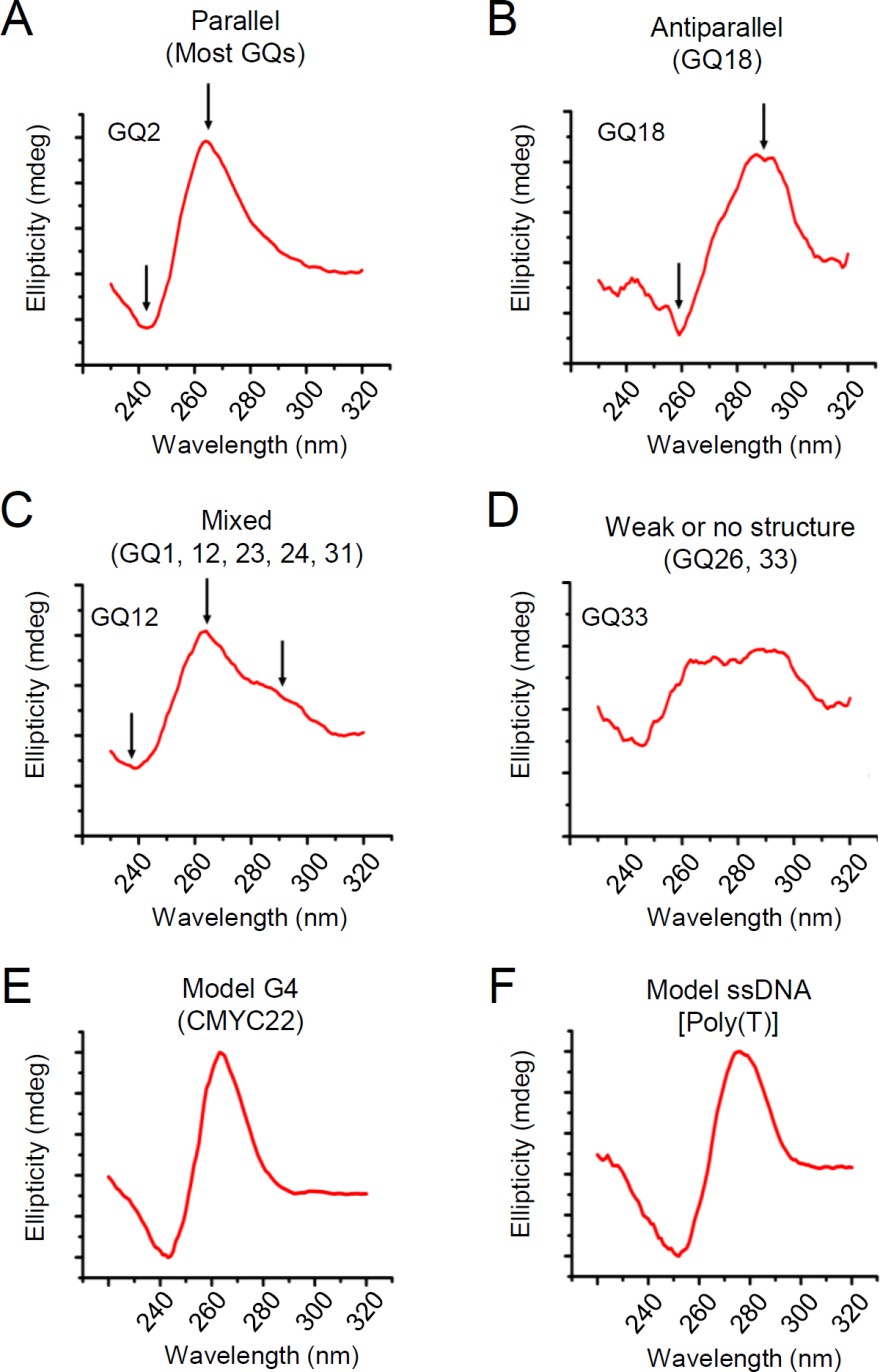
The CD spectra of the representative HCMV G4 oligonucleotides. (**A** to **D**) CD spectra of GQ2 (**A**), GQ18 (**B**), GQ12 (**C**), and GQ33 (**D**) represent the parallel, antiparallel, mixed, and weak G4 sequences, respectively. A parallel G4 (GQ2) showed a peak at 260 nm and a trough at 240 nm (**A**), whereas an antiparallel G4 (GQ18) was characterized by a peak at 290 nm and trough at 260 nm (**B**). GQ12 showed a mixed structure with a shoulder around the 290 nm region in addition to the parallel G4 peaks (**C**). In a weak G4 (GQ33), a broad shoulder was observed around the 230–280 nm region (**D**). (**E** and **F**) CD spectra of the control oligonucleotides. CD spectra of a G4-forming oligonucleotide from the promoter region of *C-MYC* (CMYC22) (as a positive control) (**E**) and a single-stranded 24mer-poly(T) [Poly(T)] (as a negative control) (**F**) are shown.

To investigate G4 formation of the identified putative G4-forming sequences, the CD spectra of the oligodeoxynucleotides corresponding to the identified sequences were measured (Fig 2; S3 Table). CD spectra can be also analyzed to classify G4 conformations—parallel, antiparallel, and mixed. Parallel conformations are characterized by a positive peak at 260 nm and a negative peak at 240 nm, while antiparallel G4s display a positive peak at 290 nm and a negative peak at 260 nm [1, 51, 52]. CD analysis revealed that most G4s (28 out of 36) displayed a prominent peak at 260 nm and a trough at 240 nm in the presence of 100 mM KCl, while only GQ18 folded into the antiparallel conformation, with the characteristic ~290 nm peak and ~260 nm trough (Figs 2 and 3A to 3B). Five G4-forming sequences, GQ1, GQ12, GQ23, GQ24, and GQ31, had mixed conformations indicated by a shoulder at 290 nm in addition to the peaks at 260 and 240 nm, and two G4-forming sequences, GQ26 and GQ33, displayed a broad plateau between the 260–280 nm region, which indicated weak G4 formation (Figs 2 and 3C to 3D). The CD spectra of a G4-forming sequence present in the promoter region of *C-MYC* (CMYC22) and a single-stranded 24mer-poly(T) [Poly(T)] were used as the positive and negative controls of G4 spectra, respectively (Fig 3E and 3F). In addition, we further analyzed the CD spectra in the presence of well-known G4 stabilization agents, 5, 10, 15, 20-tetrakis (1-methylpyridinium-4-yl) porphyrin tetra (p-toluenesulfonate) (TMPyP4) and N-methyl mesoporphyrin IX (NMM) [53, 54]. Treatment with 2× molar ratio of NMM could increase ellipticity without shifts in the ~260 nm peaks, indicating an increase in the population of G4s without changing the structure, while treatment with 2× molar ratio of TMPyP4 only showed a marginal or no increase in ellipticity and no shift in the ~260 nm peaks (Fig 2). Overall, the chemical treatment enhanced the CD ellipticity except a few cases (GQ14, GQ19, and GQ31), suggesting G4 formation can be enhanced by chemical stabilizers.

**Fig 4:**
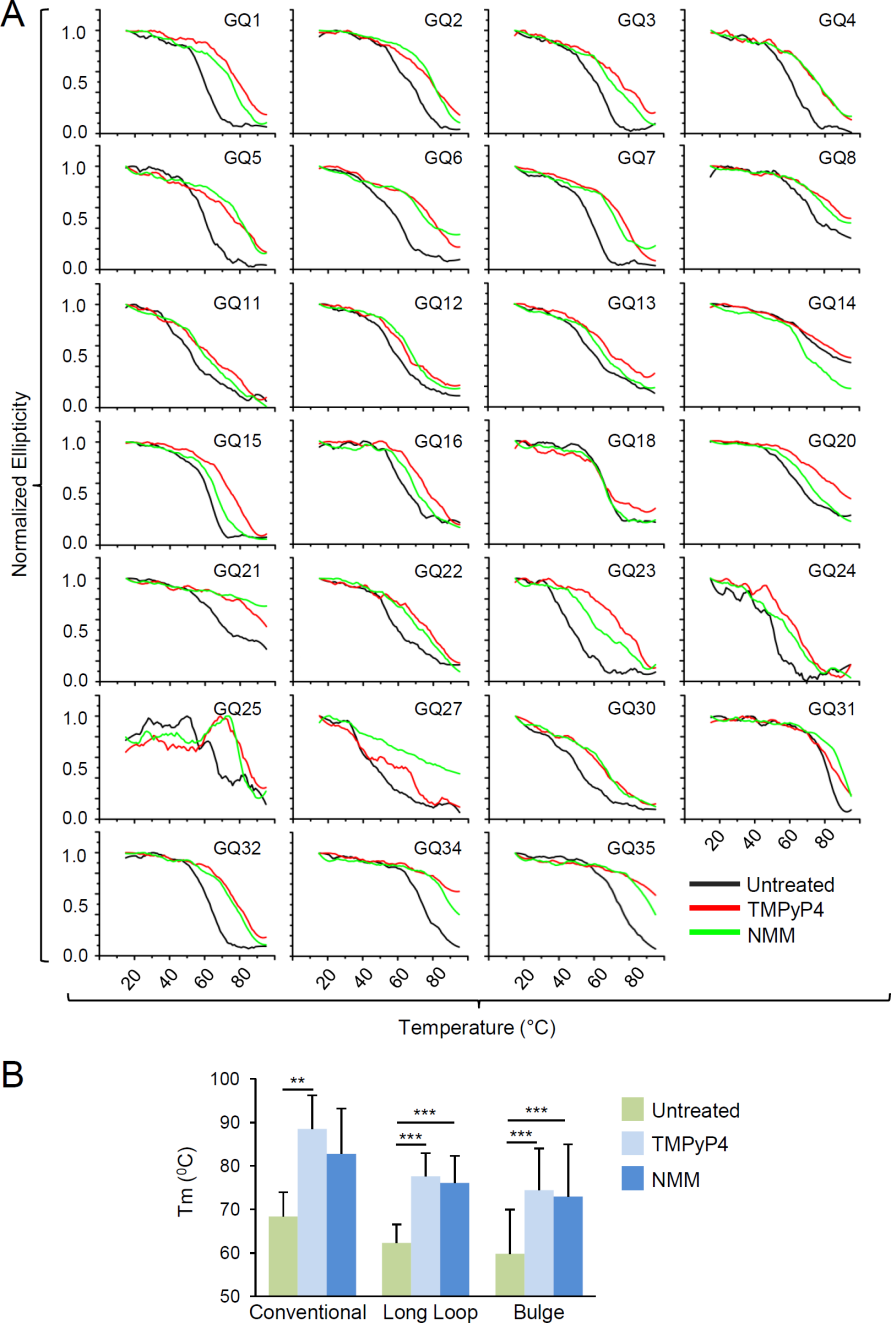
CD melting temperature (Tm) graph of HCMV G4 oligonucleotides in the absence and presence of G4 stabilizer TMPyP4 and NMM. (**A**) Comparison of CD Tm spectra of HCMV G4-forming oligonucleotides in the presence of G4 ligands, NMM and TMPyP4. Fifteen μM DNA oligos were annealed in the presence of 10 mM Tris-HCl [pH 7.5] and 100 mM KCl buffer with or without 30 μM TMPyP4 and NMM (DNA to chemical ratio 1:2). CD melting graph was calculated at 290 nm wavelength for GQ18 and at 262 nm for rest of the G4s. The data were normalized using the maximum ellipticity and smoothed using 12-point Savitzky-Golay algorithm. Note that the data of 27 GQs, which showed melting curves within the temperature range indicated, are shown. (**B**) Comparison between mean CD melting temperatures of each type of G4s in the absence or presence of TMPyP4 and NMM.

The stability of G4s was ascertained using Tm studies by monitoring the ellipticity at 262 nm, a wavelength attributed to the formation of the parallel G4, in the temperature range of 15–95°C. In the case of GQ18, ellipticity of the antiparallel G4 was examined at 290 nm. GQ26 and GQ33 were excluded from the stability analysis, because they did not display strong ellipticity at 262 nm. Tm values were determined by the first derivative method. Accordingly, those for GQ17, GQ19, GQ29/36, GQ38, and GQ28/37 were not determined since less than 50% unfolding was detected in their melting curves. Therefore, the remaining 27 G4s were further investigated for the thermal melting analysis (Table 1). Among them, 22 G4s displayed conventional sigmoid curves with Tm values of 46–80°C, indicating the complete transition from folded to unfolded phase, while the other 5 G4s (GQ8, GQ14, GQ20, GQ21, and GQ25) showed over 50% unfolding (Table 1; Fig 4A). In addition, it was also revealed that Tm values of G4s were generally enhanced in the presence of TMPyP4 and NMM except the Tm of GQ14 (Table 1; Fig 4A). We also found that conventional G4s generally had higher thermal stability than long-loop and bulged G4s, which was also consistently observed when the chemical stabilizers were treated (Fig 4B).

**Table 1:** CD melting temperature (Tm) of HCMV G4-containing oligos in the absence and presence of G4 stabilizers TMPyP4 and NMM (DNA to chemical ratio 1:2).

G4 Name	Gene Name	DNA Only	DNA + TMPyP4	DNA + NMM
GQ1	RL6	60.221	77.8237	76.6005
GQ2	UL6	65.8089	80.5812	80.944
GQ3	UL6	66.5449	76.1315	73.5733
GQ4	UL6	61.4242	76.0553	76.0598
GQ5	UL6	59.7817	83.4371	81.5559
GQ6	UL34	61.3959	79.6823	71.7368
GQ7	UL34	59.4508	78.1812	71.9152
GQ8	UL35	70.9237	84.2232	76.9593
GQ11	UL37	49.6315	61.2687	58.7847
GQ12	UL51	57.3862	62.5822	66.7411
GQ13	UL51	57.0344	65.2732	62.9627
GQ14	UL51	68.7752	73.9429	65.915
GQ15	UL51	62.9004	75.577	67.0001
GQ16	UL51	61.9147	75.3582	69.295
GQ18	UL75/76	65.88	65.0677	66.0103
GQ20	UL82	62.7413	81.0104	70.3485
GQ21	UL82	66.5796	> 95	> 95
GQ22	UL82	55.3086	81.2579	77.8253
GQ23	UL115	46.1199	77.9969	61.0188
GQ24	UL135	51.725	67.3201	63.5533
GQ25	UL138	66.7889	81.7538	80.1883
GQ27	UL142	43.6853	69.7584	> 95
GQ30	US11	50.5006	62.8887	67.2562
GQ31	US24	81.2542	87.6963	> 95
GQ32	US24	62.4511	77.7043	76.8878
GQ34	US30	74.4226	> 95	87.3144
GQ35	US30	74.661	> 95	> 95

### Context-dependent gene suppression by G4s in the viral promoter regions

**Fig 5:**
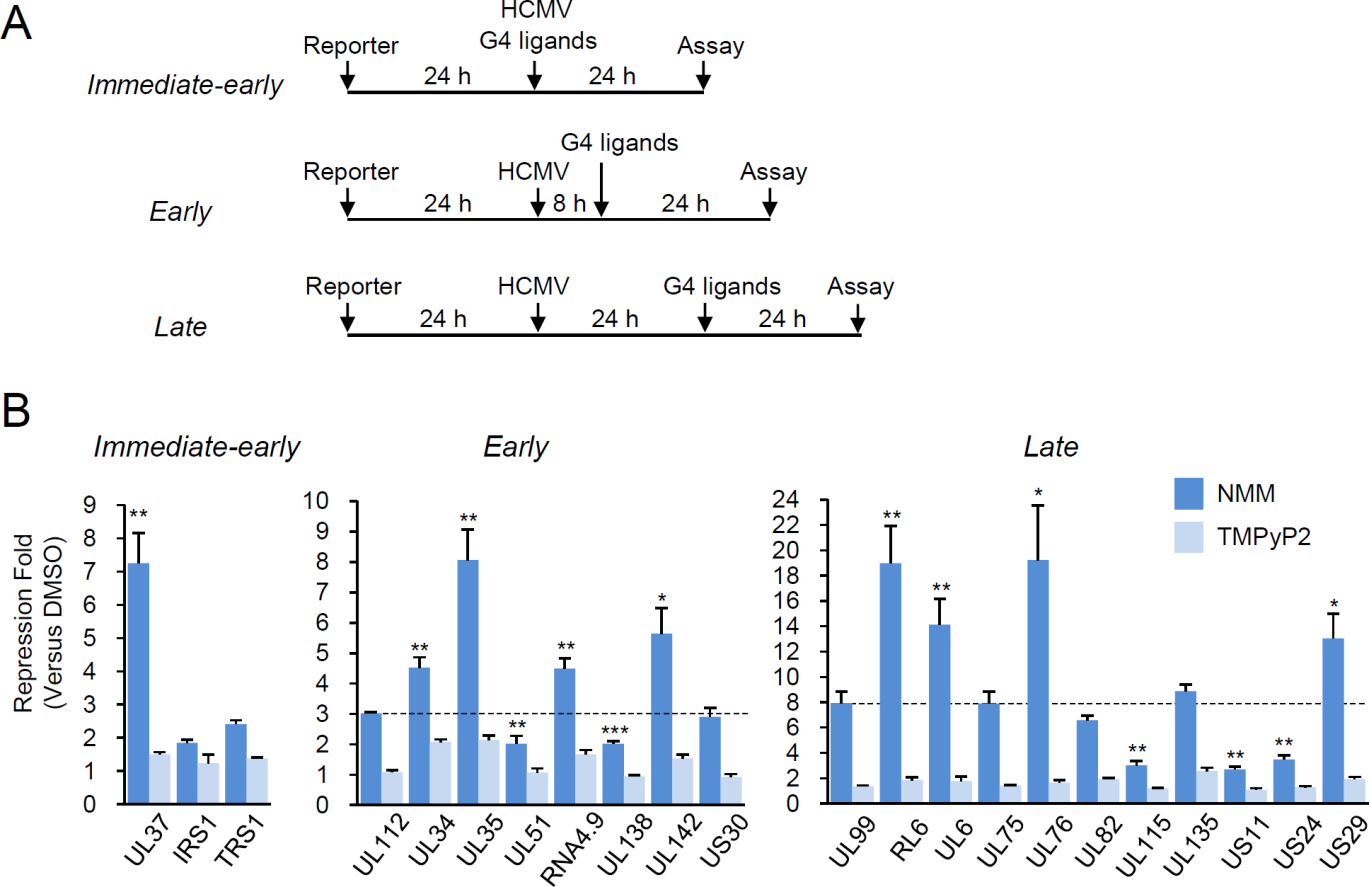
Effect of G4-binding ligands on the activity of HCMV promoters. (**A**) Schematic representation of the experimental design. HF cells were transfected via electroporation with luciferase reporter plasmid containing the viral G4-containing promoter region for 24 h. To measure the activity of promoter regions from immediate-early genes, transfected cells were infected with HCMV (Towne) at a multiplicity of infection (MOI) of 2 with treatment of DMSO (as a control) or 5 μM of NMM or TMPyP2 for 24 h. To measure the promoter activity of E and L genes, transfected cells were infected with HCMV for 8 h (for early genes) or 24 h (for late genes), and treated with DMSO or 5 μM of NMM/TMPyP2 for 24 h prior to cell harvest and luciferase assay. (**B**) Expression profiles for luciferase reporter in response to G4 ligands. The repression folds of luciferase activity by NMM or TMPyP4 treatment versus DMSO treatment obtained from triplicate samples are shown. The promoter regions of the UL112 and UL99 genes without any apparent G4 were used as controls. *P* values calculated between UL37 and IRS1/TRS1 samples, between the UL112 control gene and early genes, or between the UL99 control gene and late genes are indicated.

To explore the regulatory activity of G4s in viral gene expression during HCMV infection, we cloned 20 putative viral promoter or regulatory regions containing the 36 possible G4s into the pGL3-basic luciferase reporter plasmid. The reporter assay scheme is shown in Fig 5A. HF cells, which are fully permissive to HCMV, were transfected with luciferase reporter plasmids for 24 h prior to virus infection, and luciferase assays were performed at 24 h for immediate-early promoters, 32 h for early promoters, and 48 h for late promoters after virus infection. To analyze the effect of G4 on reporter gene expression, we treated cells with NMM, which was shown to selectively bind G4 [53, 55] and largely enhanced the G4 stability in our CD analysis, for 24 h prior to cell harvest. Meso-tetra (N-methyl-2-pyridyl) porphyrin tetrachloride (TMPyP2) was also used as a control chemical, as it did not bind G4s [56]. Both NMM and TMPyP2 did not influence HF cell viability at concentrations we used for reporter assays (S1 Fig).

The results of luciferase assays showed that among the immediate-early promoters tested, only UL37 promoter activity was significantly suppressed (by 7-fold) by NMM, but not by TMPyP2 (Fig 5B; S2 Fig). The effect of G4 stabilization on early or late promoters can be directly or indirectly affected by immediate-early or early gene expression during virus infection. Therefore, we also used UL112 and UL99 promoters, which contain about 350 bp upstream promoter sequences without any G4, as controls for analysis of early and late promoters, respectively. We found that the activities of UL112 and UL99 promoters were suppressed by NMM treatment by 3- and 8-fold, respectively, whereas they were not considerably affected by TMPyP2 (Fig 5B; S2 Fig). The suppression of UL112 and UL99 control promoters by NMM might be due to the reduced expression of immediate-early and early genes, respectively, whose expression was affected by G4 formation. Therefore, these suppression levels of control promoters were considered as basal for analysis of early and late promoters under these conditions. We found that among early promoters, UL34, UL35, RNA4.9, and UL142 promoters were significantly suppressed (by 4- to 8-fold) by only NMM and among late promoters, RL6, UL6, UL76, and US29 promoters were considerably suppressed (by 13- to 19-fold) by only NMM (Fig 5B; S2 Fig). These results of reporter assays performed in virus-infected cells demonstrated that among 20 promoters tested, only 9 promoters were significantly suppressed when G4s were stabilized by NMM. Notably, there are less correlation between the G4 stability confirmed *in vitro* (Figs 2 and 4) and G4 activity of suppressing reporter gene expression (Fig 5). These results indicate that the G4-mediated suppression of viral gene expression occurs in a promoter context-dependent manner.

**Fig 6:**
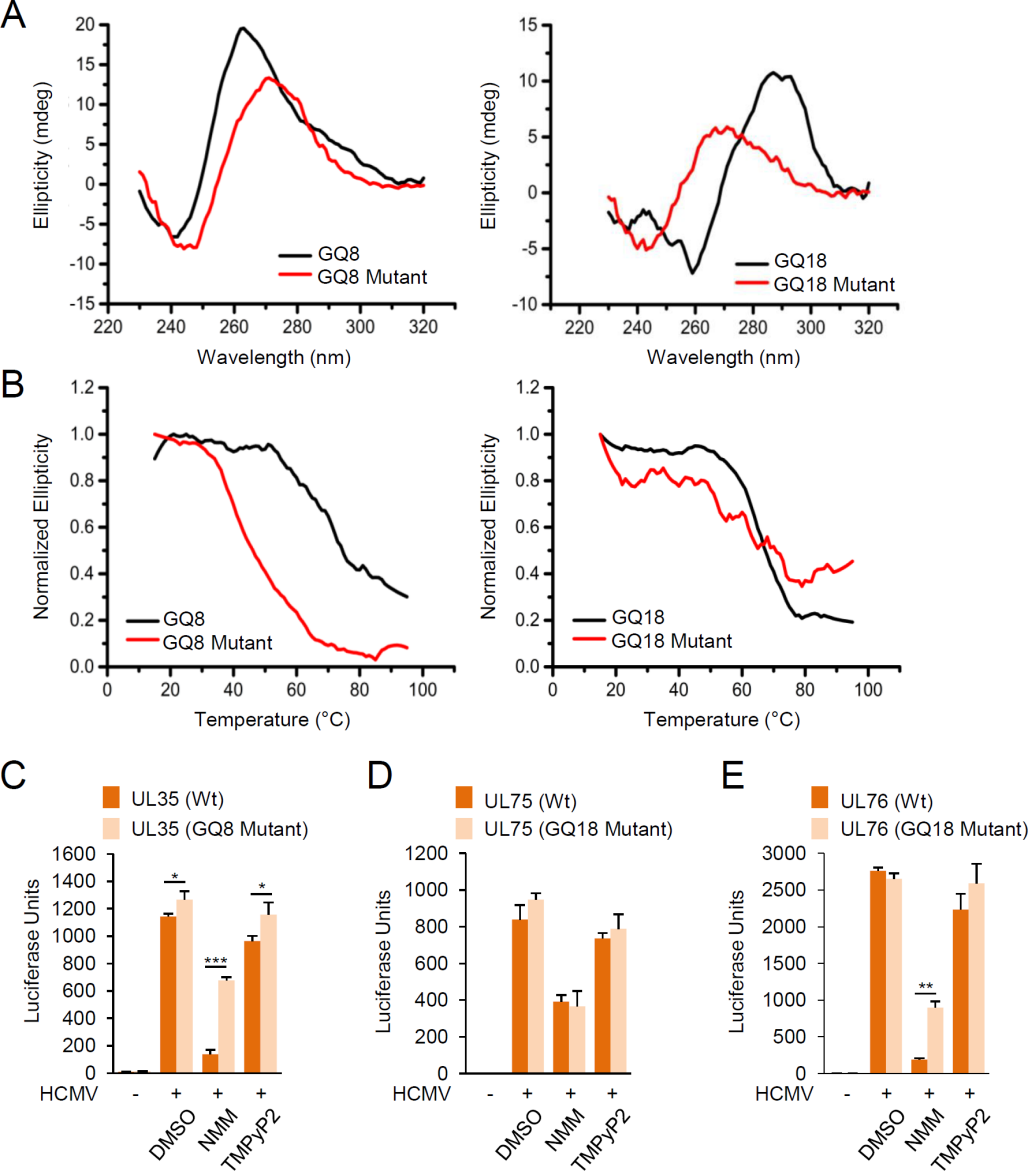
Mutational analysis of GQ8 and GQ18 for G4 stability and reporter gene suppression. (A and B) Biophysical characterization of G4 mutant oligonucleotides for GQ8 and GQ18. CD spectroscopy (A) and melting analysis (B) of GQ8 and GQ18 oligos (wild-type and G4-disrupting mutant sequences) associated with UL35 and UL75/UL76 genes, respectively, are shown. (C to E) Luciferase reporter assays demonstrating the effects of GQ8 and GQ18 mutations on promoter activities. HF cells were transfected via electroporation with reporter plasmids that expressed luciferase from the promoter regions of UL35, UL75, and UL76 containing wild-type or mutant G4 sequences. At 24 h after transfection, cells were infected with HCMV (Towne) with treatment of DMSO (as a control) or 5 μM NMM or TMPyP2 for 24 h prior to luciferase assays, which were performed at 32 h (for UL35) or 48 h (for UL75 and UL76) after virus infection. The resulting luminescence values are plotted as luciferase units.

The G4 formation of GQ8 (in UL35) and GQ18 (in UL75/76) was further analyzed using mutagenesis. GQ8 (a parallel G4) was chosen since its high propensity for gene regulation during virus infection is expected in both Tm and luciferase reporter analyses. Despite having low stability, we chose to study GQ18 (an antiparallel G4) located between the UL75 and UL76 genes, because it exerted its high suppressive effect on UL76 in cell-based reporter assays. Furthermore, the UL35 and UL76 genes are required for efficient viral growth [57, 58]. Base substitution mutations were introduced within G runs to disrupt G4 formation (S5 Table). As expected, the results of CD and Tm analyses confirmed destabilization of GQ8 and GQ18 by mutations (Fig 6A and 6B).

We introduced the same mutations of GQ8 and GQ18 into luciferase reporter plasmids containing the UL35, UL75, and UL76 promoters. HF cells were transfected with wild-type or mutant reporter plasmids, followed by HCMV infection in the absence or presence of NMM and TMPyP2. Since the basal activities of these viral promoters were very low in uninfected cells, mutation or NMM effect was only counted in virus-infected cells. The mutations within GQ8 led to only a weak increase of the UL35 promoter activity in control (DMSO-treated) cells, but they substantially mitigated the NMM-mediated suppression of UL35 expression (Fig 6C). The GQ18 mutations did not significantly affect the NMM-mediated suppression of UL75 expression (Fig 6D), consistent with the lack of NMM effect on the activity of UL75 promoter. Meanwhile, the GQ18 mutations did not change the UL76 promoter activity when cells were not treated with chemicals, but they significantly relieved the NMM-mediated suppression (Fig 6E). These results demonstrated that the NMM-stabilized, sequence-specific formation of G4s on GQ8 and GQ18 effectively suppresses UL35 and UL76 gene expression.

### Evaluation of G4-mediated gene regulation during HCMV infection

**Fig 7:**
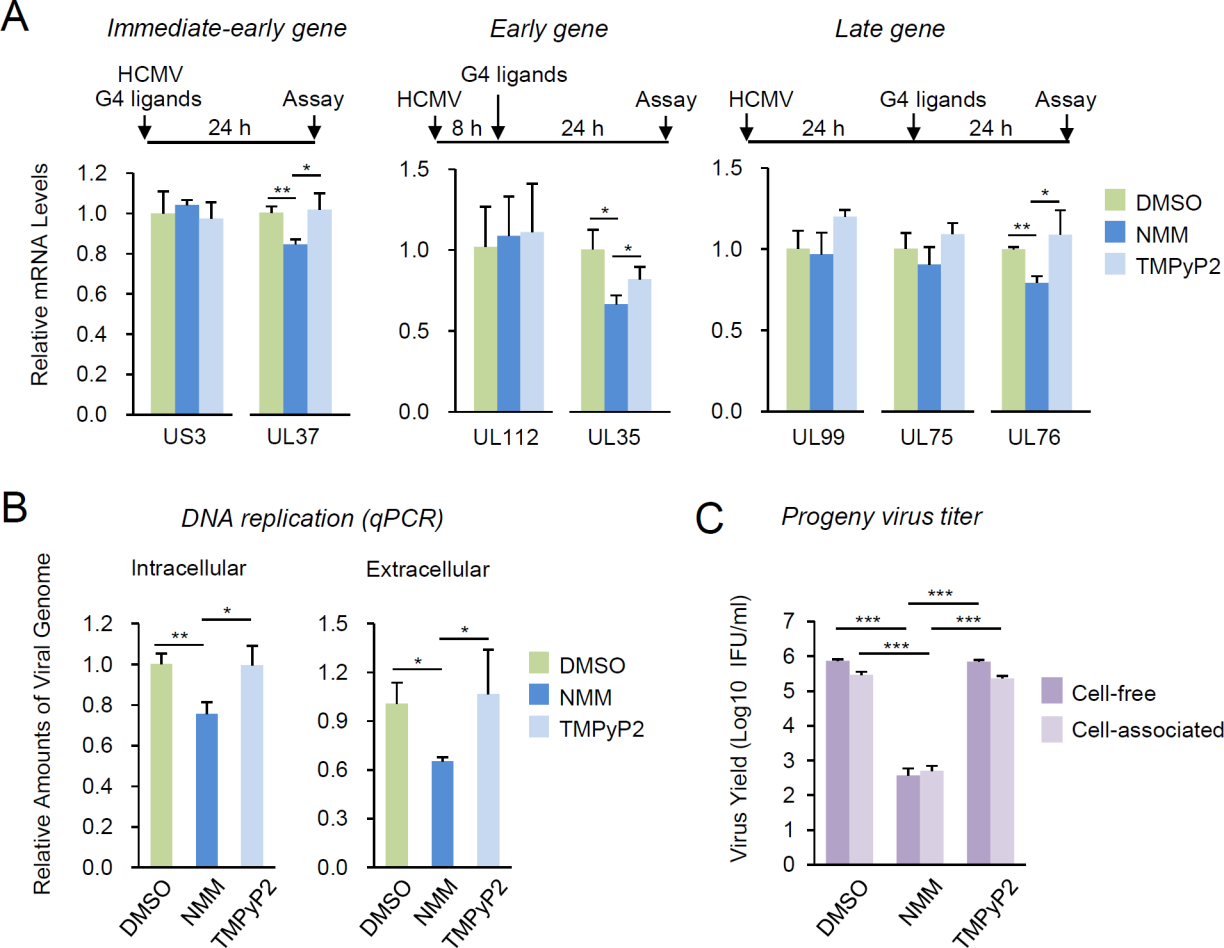
Effects of G4-binding ligands on viral gene expression, DNA replication, and production of progeny virions. (**A**) Relative mRNA levels in response to G4-binding ligands. HF cells were infected with HCMV (Toledo) at an MOI of 1, and the mRNA levels were measured by qRT-PCR at 24 h (for immediate-early genes), 32 h (for early genes) or 48 h (for late genes). To examine the effect of G4-binding ligands, cells were treated with 5 μM of NMM and TMPyP2 for 24 h prior to cell harvest. The relative mRNA levels normalized by those of β-actin are shown. (**B** and **C**) Comparison of viral DNA levels and viral titers in response to G4 ligands. HF cells were infected with HCMV (Toledo) at an MOI of 1 for 72 h. Cells and supernatants were separately collected. The viral DNA levels from the cells (intracellular) and the supernatants (extracellular) were determined by qPCR. The relative amounts of viral DNA levels normalized by those of β-actin are shown (**B**). The cell-associated and cell-free virus titers were also determined using infectious center assays (**C**).

We next evaluated the G4-mediated gene regulation during virus infection. HF cells were infected with HCMV (Toledo) for 24 h (for immediate-early gene analysis), 32 h (for early genes) and 48 h (for late genes). Virus-infected cells were untreated or treated with NMM and TMPyP2 for 24 h prior to cell harvest. The roles of GQ11, GQ8, and GQ18 in the transcription of UL37, UL35 and UL75/UL76, respectively, were assessed by measuring their mRNA levels using quantitative reverse transcription-polymerase chain reaction (qRT-PCR). The results showed that NMM treatment suppressed the UL37 mRNA level but not the US3 mRNA level (control), while the effect of TMPyP2 on these viral genes was not considerable (Fig 7A, left). Similarly, NMM treatment suppressed the UL35 mRNA level but not the UL112 mRNA level (control), while TMPyP2 did not show a considerable effect (Fig 7A, center). NMM also significantly suppressed the UL76 mRNA level, but not the UL99 (control) and UL75 mRNA levels, whileTMPyP2 did not affect these late genes (Fig 7A, right). These results suggest that G4 stabilization on GQ11, GQ8, and GQ18 by ligand binding indeed suppresses the transcription of UL37, UL35, and UL76, respectively.

We also compared the levels of viral DNA with or without ligands from the infected cells and from the culture supernatant. The results of qPCR indicated that NMM treatment reduced both intracellular and extracellular viral DNA levels to 75 and 65%, respectively, of those in dimethyl sulfoxide (DMSO)-treated cells, while TMPyP2 did not significantly affect the viral DNA levels (Fig 7B). When we measured the infectivity of newly produced progeny virions by infectious center assays, the infectivity of both cell-associated virions and those released into the culture medium was more significantly reduced by treatment of NMM, but not by TMPyP2 (Fig 7C). The antiviral effect of G4-stabilizing ligands on herpes simplex virus-1 (HSV-1) has been reported [41, 59]. Consistently, we also found that NMM treatment suppresses the growth of HSV-1 (S3 Fig).

**Fig 8:**
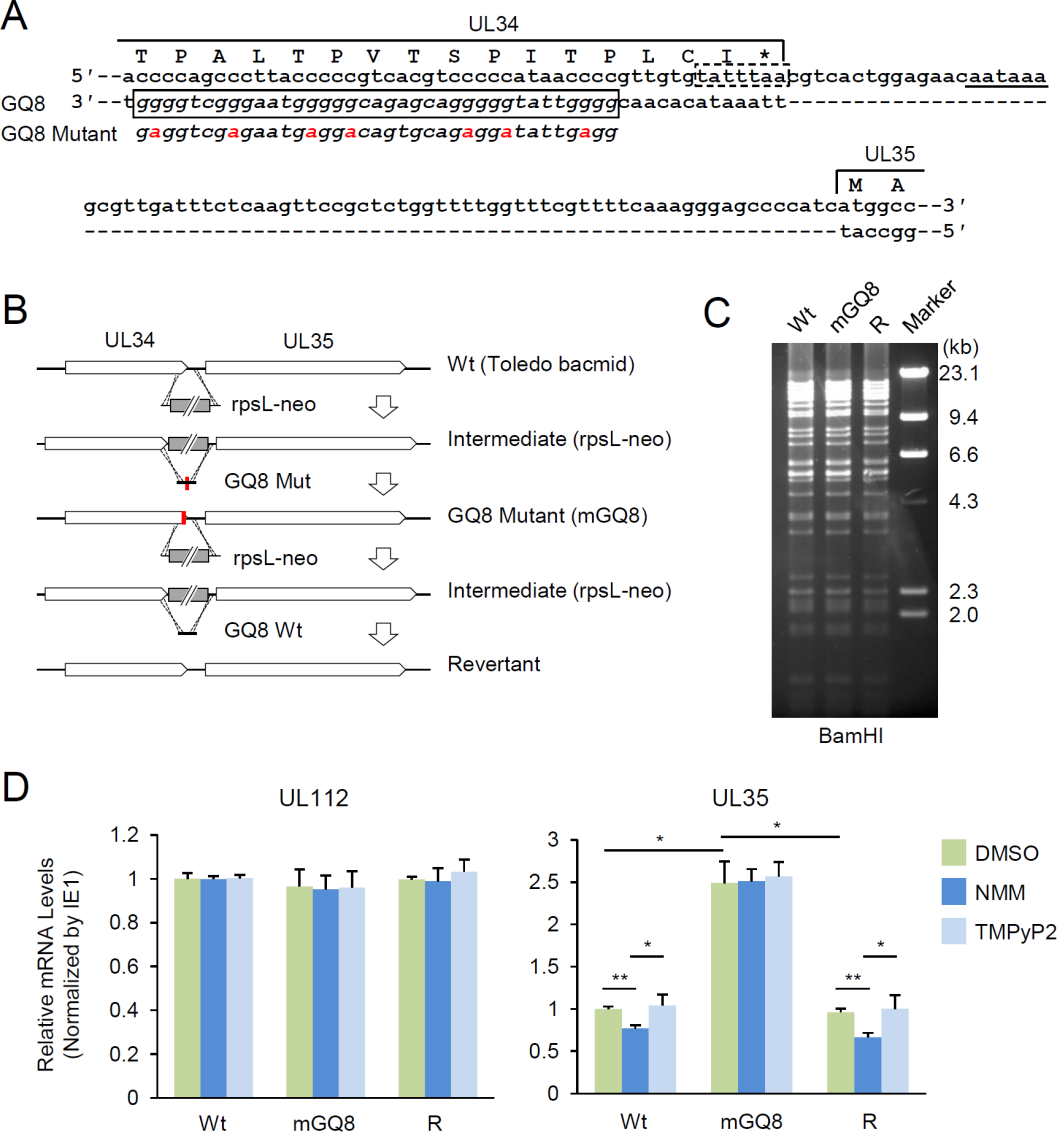
Analysis of the GQ8 mutant virus. (**A**) Schematic representation of the regulatory region of the UL35 gene. Positions of the GQ8 (solid box) and putative TATA box (dashed box) for UL35 are indicated. The predicted polyadenylation signal for UL34 is underlined. Mutations introduced into GQ8 to disrupt G4 formation are indicated in red. The parts of UL34 and UL35 ORFs are indicated with amino acid sequences. (**B**) Graphical representation of various mutants and revertant viruses generated for the study of GQ8. The HCMV (Toledo) bacmid containing mutant GQ8 (mGQ8) and its revertant were produced using a counter-selection BAC modification kit (Gene Bridges) (see Materials and Methods). (**C**) Gel profiles showing restriction patterns of HCMV bacmid constructs. Wild-type (Wt), GQ8 mutant (mGQ8), and revertant (R) bacmids were digested with BamH1 and subjected to pulse-field gel electrophoresis. No apparent alteration of restriction fragment patterns was found in mGQ8 and revertant bacmids. (**D**) Effect of G4 ligands on UL35 transcription in wild-type, GQ8 mutant, and revertant viruses. HF cells were infected with recombinant HCMV [wild-type (Wt), GQ8 mutant (mGQ8), or revertant (R)] at an MOI of 1 and treated with DMSO, 5 μM of NMM, or TMPyP2 for 24 h prior to cell harvest at 32 h after HCMV infection. The mRNA levels of UL112 and UL35 were measured by qRT-PCR. The relative mRNA levels normalized by those of β-actin and IE1 are shown as graphs.

Finally, the suppressive role of GQ8 in UL35 expression was investigated by producing a recombinant virus containing G4-disrupting mutations. The mutations introduced did not affect the coding potential of the overlapping UL34 gene (Fig 8A). The HCMV (Toledo) bacmids containing GQ8 mutant (mGQ8) and its revertant were produced by bacmid mutagenesis using the counter-selection marker rpsL-neo, and recombinant viruses were grown in HF cells after electroporation of the bacmid DNAs (Fig 8B and 8C). The overall growth of the GQ8 mutant virus in HF cells was not significantly altered compared to the wild-type virus. However, when HF cells were infected with recombinant viruses with or without NMM treatment and the mRNA levels of UL35 and UL112 (control) were compared by qRT-PCR, we found that the UL35 mRNA level was increased in mGQ8 mutant virus infection compared to wild-type and revertant virus infection, and that NMM-mediated suppression of UL35 expression was observed in wild-type and revertant virus infection, but not in GQ8 mutant virus infection (Fig 8D). These results using this GQ8 mutant virus incapable of G4 formation in the UL35 promoter region confirm the suppressive role of G4 formation in gene expression during virus infection.

## Discussion

G-rich sequences capable of forming G4 structures are present in the genome of diverse organisms and have emerged as a therapeutic target in recent years [17]. Structural and functional studies of G4s have been attempted largely in higher order organisms including humans [12, 20]. However, getting a complete picture of all G4 functions in a genome is difficult because of the large number of putative G4-forming sequences in higher organisms. In this study using the 235-kb HCMV genome, we identified 36 putative G4-forming sequences (denoted as GQs in the HCMV genome) in the putative promoter or gene regulatory regions. We proved that many of the putative G4-forming sequences could indeed form stable structures using CD spectroscopic and Tm analysis. Importantly, by evaluating the gene regulation by G4s in HF cells using intact or G4-mutant promoters, we discovered that the gene suppression by a specific G4 was promoter context-dependent. Furthermore, we provided evidence for the G4-mediated gene suppression during HCMV infection by employing a mutant virus incapable of forming a G4 (from GQ8) in the UL35 promoter.

An earlier study has demonstrated that the density of potential G4-forming sequences was relatively high in herpesvirus genomes compared to that in human and mouse genomes [47]. In this study we identified more G4-forming sequences by applying the new search scheme that can detect the nonconventional (bulged and long-loop) G4s. By this approach, we found that HCMV contains the high content of the nonconventional G4 compared to the human. Based on our *in vitro* Tm analysis, we found that the stability as represented by high Tm value was generally higher in conventional G4s than in bulged or long-loop G4s (Fig 4B). The functional difference of different G4 types has not been elucidated. However, our findings that conventional G4s have higher stability than bulged or long-loop G4s are consistent with the earlier findings that thermal stability of G4s can be affected by the presence of a loop or bulge [60]. We also found that G4-forming sequences were distributed throughout the entire HCMV genome, and among them, 36 G4s were associated with 20 genes; immediate-early (3), early (7), and late (10) genes. These regulatory G4s were unbiasedly found in both positive and negative strands relative to their downstream genes. Using CD spectroscopy, we also found that most identified G4s formed parallel G4s, while only one formed an antiparallel G4, suggesting that parallel G4s might be more prevalent in the HCMV genome than antiparallel G4s.

By Tm analysis, we found that NMM and TMPyP4 generally increased the Tm of most stable G4s. NMM, which binds to parallel G4 [61], did not affect the stability of GQ18, an antiparallel G4, in CD analysis. However, in our cell-based reporter assays, we observed that NMM treatment of cells resulted in a severe suppression of UL76 gene expression possibly by affecting GQ18 in the promoter within the cells. In this sense, it is notable that the parallel conformation is favored in double-stranded DNA irrespective of the sequence as reported previously [62], suggesting that G4 structure can be converted in the cells. Indeed, we observed that GQ18 folded into a parallel G4 in NaCl solution (S4 Fig). Therefore, we inferred that GQ18, in the context of the viral genome, might form a parallel G4 within the cell. We observed that GQ26 and GQ33, which did not show any characteristic CD spectra and, thereby, were considered to form weak structures, showed medium-range suppression in cell reporter assay in the presence of NMM (UL142 and US29, respectively; Fig 5B). This result suggest even weak G4 structures can possibly affect the gene expression when they form in cells.

Overall, our analyses of physicochemical properties of G4s and the HCMV promoter activity in HF cells revealed that the gene expression is suppressed by G4s, but the suppression levels are not correlated to the *in vitro* stability of G4. Therefore, it is likely that the G4 effect on gene expression is dependent on the promoter context rather than on the G4 stability observed in *in vitro* analysis. For example, although GQ34 and GQ35 belonging to US30 showed high *in vitro* biophysical stability (Fig 4; Table 1), their effect on the cell-based reporter study was below the set threshold value (Fig 5B). This suggested that the G4 formation in a G-rich sequence and their functionality within the cell may be influenced by many factors including neighboring sequences and cellular proteins associated with them. Indeed, among the 20 potential G4-containing promoters, only 9 promoters were affected by G4 formation. This emphasized the importance of using the whole promoter regions and the cell-based assays when assessing a specific role of G4 in gene regulation.

In addition, using the cell-based reporter analysis of G4 mutant promoters, we confirmed that the NMM-stabilized, sequence-specific G4 formation of GQ8 (in UL35) and GQ18 (in UL76) indeed suppressed the reporter gene expression. Consistently, the mRNA levels of UL35 and UL76 were reduced in HCMV-infected cells by NMM treatment. Our study further addressed the role of G4 formation (from GQ8) in the UL35 promoter in the context of virus infection by producing a recombinant virus with mutant GQ8. The GQ8 is located adjacent to the A/T-rich sequence that appears to act as a TATA-box for the UL35 gene [63]. Our study is the first report providing *in vivo* evidence for G4-mediated gene regulation during herpesvirus infection. We found that the suppressive effect of G4 on promoter activity was mostly seen in the presence of a G4-stabilizing ligand, suggesting that the G4 formation is dynamically regulated within the cells. It is also notable that the effect of GQ8 mutations on the UL35 promoter without NMM treatment was bigger in the viral genome (Fig 8D) than in the reporter plasmid (Fig 6C). This suggests that the GQ8 may more effectively form a G4 in the viral genome than in the plasmid without chemical stabilization, suggesting a possible regulatory effect of chromatinization on G4 formation. This further highlights the importance of analyzing the activity of G4-forming sequences in the viral genome context.

An intriguing question arising from the present study is why HCMV has several G4-forming sequences in its promoters, if they suppress viral gene expression. Given that the G4 formation in the HBV pre-S2/S promoter region has a positive effect on gene transcription [44], we think that gene suppression by G4s in HCMV genome is not fortuitous, but rather the result of viral genome evolution. It is tempting to speculate that the G4 formation plays a role in establishing latent infection of HCMV. Notably, our results showed that NMM effectively inhibited HCMV growth in HF cells without affecting cell viability compared to other ligands tested. We do not think that the suppression of gene expression via G4 formation fully accounts for the NMM-mediated inhibition of HCMV growth. We also found G4-forming sequences in the *ori*Lyt region and the repeated regions of HCMV genome. Therefore, NMM-meditated regulation of G4s may also affect DNA replication. The detailed molecular mechanism other than gene suppression, by which NMM inhibits HCMV growth, awaits further investigation.

Our study provides a platform for assessing G4 functions for gene expression in viral genomes. Through computational search, different types of G4-forming sequences such as conventional, long-loop, and bulged can be predicted from the genome sequences. The formation of parallel and antiparallel G4s and their stability can be determined by CD spectroscopy and Tm analyses. However, the role of a G4 in gene expression should be addressed using cell-based assays in the context of viral genome sequence, as the G4 formation as well as its effect on promoter activity can be influenced by G4-neighboring sequences and associated cellular factors. Overall, our results point to a relevant physiological role of G4s in controlling HCMV viral gene expression and provide a new insight into understanding gene regulation by G4 structures. We believe that further genome-wide analyses of G4s in different viruses or more organisms containing more complex genome structures will help us to establish a general link between G4s in virus genomes and their involvement in the virus life cycle.

## Materials and methods

### Chemicals

NMM and TMPyP2 were purchased from Frontier Scientific. TMPyP4 tosylate was bought from Abcam. BRACO19 and Pyridostatin (PDS) were purchased from Sigma-Aldrich.

### Bioinformatics analysis

The HCMV Toledo strain (GenBank: GU937742.1) was used in the genome-wide prediction of putative G4-forming sequences within −500 to +100 regions relative to the translation initiation site of the genes. The TATA boxes have been studied for a limited number of the genes. Therefore, for most genes the possible locations of TATA boxes were predicted using the SoftBerry promoter/functional motifs prediction server (http://www.softberry.com/berry.phtml). Three different types of G4-forming sequences were considered for the prediction: (i) conventional G4s [G(3–6), N(1–7), G(3–6), N(1–7), G(3–6), N(1–7), G(3–6)], (ii) long-loop G4s [single loop; G(3–6), N(8–50), G(3–6), N(1–2), G(3–6), N(1–2), G(3–6) and other two combination for two different loops], (iii) bulge-containing G4s [single bulge; GG(ATC)G, N(1–7), G(3–6), N(1–7), G(3–6), N(1–7), G(3–6) and other three combinations for the remaining three G runs]. All the loop regions of long-loop G4s were analyzed using the Mfold secondary structure prediction server (http://unafold.rna.albany.edu) to calculate secondary structure-forming ΔG values, and only the long-loop G4s that held considerably low ΔG values were selected for further analysis. Finally, we selected those G4s which were conserved in the genome of the HCMV Merlin strain. After filtering through all the selection criteria, we selected 36 possible G4-forming sequences in the putative regulatory regions from the HCMV genome for further studies.

### CD spectroscopy and Tm analysis

Oligonucleotides used for the CD spectroscopy are described in S3 Table. CD spectroscopy was performed on a Jasco J-810 spectroscopy fitted with a Peltier temperature controller. The oligonucleotides were dissolved at a concentration of 15 μM in a buffer containing 100 mM KCl and 10 mM Tris-HCl [pH 7.5] followed by denaturation at 95°C for 5 min and annealing at room temperature over a period of 2 h. For studies with G4-binding ligands, pre-formed GQs were treated with 30 μM NMM or TMPyP4 for a DNA-to-ligand ratio of 1:2. CD spectra were measured at 25°C as the average of 3 accumulations between 230–320 nm, with a response time of 2 sec, scanning speed of 100 nm/min, and data pitch of 1 nm. CD melting curves were recorded between 15–95°C at a wavelength of 262 nm for all nucleotides except GQ18, for which the data were recorded at 290 nm. After subtracting the spectrum of buffer only from all samples, the data were normalized to the maximum ellipticity. The first derivative of the melting curve was plotted and fitted using inbuilt functions in Sigma-Plot 12.5.

### Construction of the reporter plasmids containing the putative viral promoter or regulatory regions

The viral regulatory regions containing the identified G4s were amplified by PCR as the NheI-BglII or KpnI-BglII fragments (300 to 560-bp) from the HCMV (Towne strain) bacmid DNA. These amplified regions contained the minimal putative regulatory regions. If G4s were identified upstream of the predicted TATA boxes, the amplified regions included upstream G4-TATA box-ATG (the translation initiation site). If G4s were predicted between the putative TATA boxes and ATG, or in the regions without any putative TATA box-like sequences, at least 300-bp regions including TATA box-G4-ATG or G4-ATG were amplified. The primer sets used for PCR are given in S4 Table. The amplified DNAs were digested with restriction enzymes and cloned into a promoter-less firefly luciferase vector pGL3-basic (Promega). Luciferase reporter plasmids containing the UL112 [64] and UL99 [65] promoters were obtained from Thomas Stamminger (Ulm University Medical Center, Germany) and Gary Hayward (John Hopkins Medicine, USA), respectively.

### Cell culture, virus, and electroporation

Primary HF cells (American Type Culture Collection; ATCC PCS-201-010) were grown in Dulbecco’s modified Eagle’s medium (DMEM) supplemented with 10% fetal bovine serum, penicillin (100 U/mL), and streptomycin (100 μg/mL) in a 5% CO_2_ humidified incubator at 37°C. The HCMV Towne strain and recombinant HCMV (Toledo strain) that was prepared from the HCMV (Toledo) bacmid were previously described [66]. HF cells were transiently transfected with reporter plasmids via electroporation. Electroporation was performed at 1,300 V for 30 ms using a Microporator MP-100 (Digital Bio) according to the manufacturer’s instructions.

### Luciferase reporter assays

Cells were harvested and lysed by three freeze-thaw steps in 100 μL of lysis buffer (25 mM Tris-Cl and 1 mM dithiothreitol). Twenty microliters of cell lysates were incubated with 350 μL of reaction buffer A (25 mM glycyl-glycine [pH 7.8], 5 mM ATP [pH 7.5], 4 mM egtazic acid [pH 8.0], and 15 mM MgSO_4_) and mixed with 100 μL of 0.25 mM luciferin (Sigma-Aldrich) in the reaction buffer A. The luciferase units were measured using a TD-20/20 luminometer (Turner Design). The assays were performed in triplicate.

### Site-directed mutagenesis

To introduce mutations that disrupted G4 formation within the viral promoters of UL35 (GQ8) and UL75/UL76 (GQ18), PCR reactions were performed according to the Stratagene QuikChange site-directed mutagenesis protocol. The following primers were used: 5′-ACTCCAGCTCTTACTCCTGTCACGTCTCCTATAACTCCGT-3′ (GQ8 forward) and 5′-ACGGAGTTATAGGAGACGTGACAGGAGTAAGAGCTGGAGT-3′ (GQ8 reverse), and 5′-GTGTAGCGCTACGAGTTACAAAAGTCG-3′ (GQ18 forward) and 5′-CGACTTTTGTAACTCGTAGCGCTACAC-3′ (GQ18 reverse). All mutant constructs were verified by sequencing.

### Quantitation of viral DNAs

Total DNA was isolated from infected cells or culture medium using the QIAamp DNA Mini kit (Qiagen) and eluted in 100 μL of sterile water. Five microliters of elute was used for qPCR to measure the amount of viral DNA using the Power SYBR Green PCR Master Mix and QuantStudio Real-Time PCR System (Applied Biosystems). The primers to amplify the UL75 gene were used for viral DNA quantitation: 5′-GCAAAAGGCGCAGTTTTCTA-3′ (forward) and 5′-TCCTACCCTGTCTCCACAC-3′ (reverse). The primers for β-actin amplification were used for normalizing the threshold cycle (C_t_) values: 5′-TCACCCACACTGTGCCCATCTACCA-3′ (forward) and 5′-CAGCGGAACCGCTCATTGCCAATGG-3′ (reverse).

### Quantitation of viral mRNAs

Total RNA was extracted from virus-infected cells (2 × 10^5^) using TRI reagents (Molecular Research Center) and MaxTract High Density (Qiagen). QuantiTect Reverse Transcription kit (Qiagen) was used to generate cDNAs. qRT-PCR was performed using the Power SYBR Green PCR Master Mix and QuantStudio Real-Time PCR System. The following primers were used: 5′-ATAAGCGGGAGATGTGGATG-3′ (IE1 forward), 5′-TTCATCCTTTTTAGCACGGG-3′ (IE1 reverse), 5'-AGTCCGTTTGAGTCATCCGT-3' (UL37 forward), 5'-AATCGCGGACACATGTCTTG-3' (UL37 reverse), 5′-TTGCAGCTACTGACGCAACT-3′ (UL35 forward), 5′-TTCTCCTGCTCTTCGTCCTC-3′ (UL35 reverse), 5′-GCAAAAGGCGCAGTTTTCTA-3′ (UL75 forward), 5′-TCCTACCCTGTCTCCACCAC-3′ (UL75 reverse), 5′-AAGCACCTGGACATCTACCG-3′ (UL76 forward), 5′-TCCGCCGACTTAATCGTACT-3′ (UL76 reverse), 5′-GAGGACAAGGCTCCGAAAC-3′ (UL99 forward), 5′-CTTTGCTGATGGTGGTGATG-3′ (UL99 reverse), 5′-GGTGCGTTACTTCTACCCATT -3′ (UL112 forward), 5′-TTAGGTCCTCGCGACGCTGCT -3′ (UL112 reverse), 5'-CTGGATGTGGTGGTATCGGA-3' (US3 forward), 5'-TGTTTCTCGGTGAAGTTGCC-3' (US3 reverse), 5′-AGCGGGAAATCGTGCGTG-3′ (β-actin forward), and 5′-CAGGGTACATGGTGGTGCC-3′ (β-actin reverse).

### Titration of progeny virions

Virus titers were determined by infectious center assays. HF cells (1 × 10^5^) were seeded into 24-well plates and incubated for 24 h before infection. Ten-fold serial diluted viral stocks (cell-associated or culture supernatants) (10^−1^ to 10^−3^) were added to each well and incubated for 1 h, followed by replacement with 1 mL fresh medium. At 24 h, cells were fixed with 500 μL of methanol at 4°C for 10 min. Cells were washed three times with phosphate-buffered saline (PBS) and incubated with anti-IE1 rabbit polyclonal antibody (PAb) in PBS at 37°C for 1 h, followed by incubation with phosphatase-labeled anti-rabbit immunoglobulin (IgG) in PBS at 37°C for 1 h. Finally, the cells were treated with 200 μL of AP buffer (100 mM Tris-HCl, 100 mM NaCl, and 5 mM MgCl_2_) mixed with 5-bromo-4-chloro-3-indolyl phosphatase/nitro blue tetrazolium (BCIP/NBT, Millipore) at a 1:1 ratio. The IE1-positive cells were counted in five separate fields per well under a light microscope.

### Bacmid mutagenesis

The Toledo-bacmid encoding the UL35 (mGQ8) genes were generated by using a bacterial artificial chromosome (BAC) modification kit (Gene Bridges). Briefly, the rpsL-neo cassettes flanked by homology arms with 100 nucleotides of the region upstream and downstream of the target site (UL35 GQ8) were amplified using the following primer sets: 5′-CGGGTCGCCGCGACCCCCTCACCTTCAGTCACCCCAGCCCTTACCCCCGTGGCCTGGTGATGATGGCGGGATCG-3′ and 5′-TTATTGTTCTCCAGTGACGTTAAATACACAA CGGGGTTATGGGGGACGTGTCAGAAGAACTCGTCAAGAAGGCG-3′. The amplified rpsL-neo fragments were purified and introduced into *E*. *coli* DH10B containing wild-type Toledo-bacmids for recombination via electroporation using Gene Pulser II (Bio-Rad). The intermediate Toledo-bacmid construct containing the rpsL-neo cassette was selected on Luria Bertani (LB) agar plates containing kanamycin. Next, the mGQ fragments for replacing the rpsL-neo cassette were generated by annealing two single-stranded oligonucleotides. The following oligonucleotide sets were used: 5′-CGGGTCGCCGCGACCCCCTCACCTTCAGTCACTCCAGCTCTTACTCCTGTCACG TCTCCTATAACTCCGTTGTGTATTTAACGTCACTGGAGAACAATAA-3′ and 5′-TTATT GTTCTCCAGTGACGTTAAATACACAACGGAGTTATAGGAGACGTGACAGGAGTAAGAGCTGGAGTGACTGAAGGTGAGGGGGTCGCGGCGACCCG-3′ [for UL35 (mGQ8)]. The annealed oligonucleotides were recombined into the Toledo-bacmid DNAs containing the rpsL-neo cassette, and the *E*. *coli* cells containing the UL35 (mGQ8) Toledo-bacmid were selected on LB plates containing streptomycin. The mutated regions were amplified by PCR and sequenced to verify the desired mutations. To generate the revertant Toledo-bacmid from the mutant, the wild-type G4 fragments were also generated by annealing using the following oligonucleotide sets: 5′-CGGGTCGCCGCGACCCCCTCACCTTCAGTCACCCCAGCCC TTACCCCCGTCACGTCCCCCATAACCCCGTTGTGTATTTAACGTCACTGGAGAACAATAA-3′ and 5′-TTATTGTTCTCCAGTGACGTTAAATACACAACGGGGTTATGGGGGA CGTGACGGGGGTAAGGGCTGGGGTGACTGAAGGTGAGGGGGTCGCGGCGACCCG-3′. These fragments were inserted into the mutant Toledo-bacmid by homologous recombination as described above.

### Statistical analysis

Statistical significances are determined using the Student’s *t*-test and indicated by *p*-values < 0.05 (*), < 0.01 (**), and < 0.001 (***).

## Supporting information

S1 Appendix Putative G4-forming sequences predicted in the HCMV (Toledo strain) genome. (XLSX)

S1 Fig Effect of G4-binding ligands on HF cell viability. Comparison of cell viability of human fibroblast (HF) cells in the presence of various G4 ligands upon HCMV infection. Intact HF cells (**A**) or cells infected with HCMV(Towne) at an MOI of 1 (**B**) were treated with DW, DMSO, or G4-binding ligands [NMM (10 μM), TMPyP4 (10 μM), TMPyP2 (10 μM), BRACO19 (5 μM), or pyridostatin (PDS) (10 μM] as indicated for 72 h. Cell viability was measured using 3-(4,5-dimethylthiazol-2-yl)-2,5-diphenyltetrazolium bromide (MTT) assays. The results shown are averages of triplicates with error bars. Statistical significance of samples (relative to D.W. controls) was determined using the *t*-test, and p-values < 0.05 (*), 0.01 (**), and 0.001 (***) are indicated.
(TIF)

S2 Fig The luciferase units obtained from the luciferase assays. Comparison of raw luminescence values from luciferase assays with specific gene regulatory region-containing reporter constructs in the presence of G4 ligands. The luciferase units obtained from luciferase reporter assays in Fig 5B are shown here. Statistical significance of samples (relative to DMSO controls) was determined using the *t*-test, and p-values < 0.05 (*), 0.01 (**), and 0.001 (***) are indicated. (A) Immediate-early genes. (B) Early genes. (C) Late genes. D, DMSO; N, 5 μM of NMM; T, 5 μM of TMPyP2.
(TIF)

S3 Fig Dose-dependent effect of NMM on HSV-1 growth. HF cells were infected with HSV-1 at an MOI of 1 and treated with DMSO (as a control) or increasing concentrations of NMM or TMPyP2. At 24 h after infection, the culture supernatants were collected at 24 h and virus titers were determined using plaque assays in Vero cells.
(TIF)

S4 Fig Comparison of CD spectra of GQ18 in K^+^ and Na^+^ buffers. Fifteen μM DNA GQ18 oligonucleotides were annealed in the presence of 10 mM Tris-HCl [pH 7.5] and 100 mM NaCl or KCl buffer. The CD spectrum in NaCl buffer (black) is compared with that obtained in KCl buffer (red).
(TIF)

S1 Table Sequences, types, and G4 strand positions of the putative 38 GQs (GQ1~GQ38) identified in the putative regulator regions of HCMV genome (-500 and +100 with respect to the translation initiation sites). (PDF)

S2 Table Positions of the 38 GQs (GQ1~GQ38) in relation to TATA boxes and translation initiation sites of corresponding genes. (TIF)

S3 Table Oligonucleotides used in CD spectroscopy and melting experiments. The G4-forming sequences for the analyzed 36 GQs are indicated in blue.
(TIF)

S4 Table Primers used for cloning plasmid vectors for cell-based luciferase assays. (TIF)

S5 Table Wild-type and mutant oligos used for CD spectroscopy analysis of GQ8 (UL35) and GQ18 (UL75/76). Mutation sites are indicated by red characters.
(TIF)
