# Supplementary figures and images for: Genome-wide analysis of regulatory G-quadruplexes affecting gene expression in human cytomegalovirus
Source: PLoS Pathog. 2018 Sep 28;14(9):e1007334. doi: 10.1371/journal.ppat.1007334 (PMC6179306; doi:10.1371/journal.ppat.1007334)

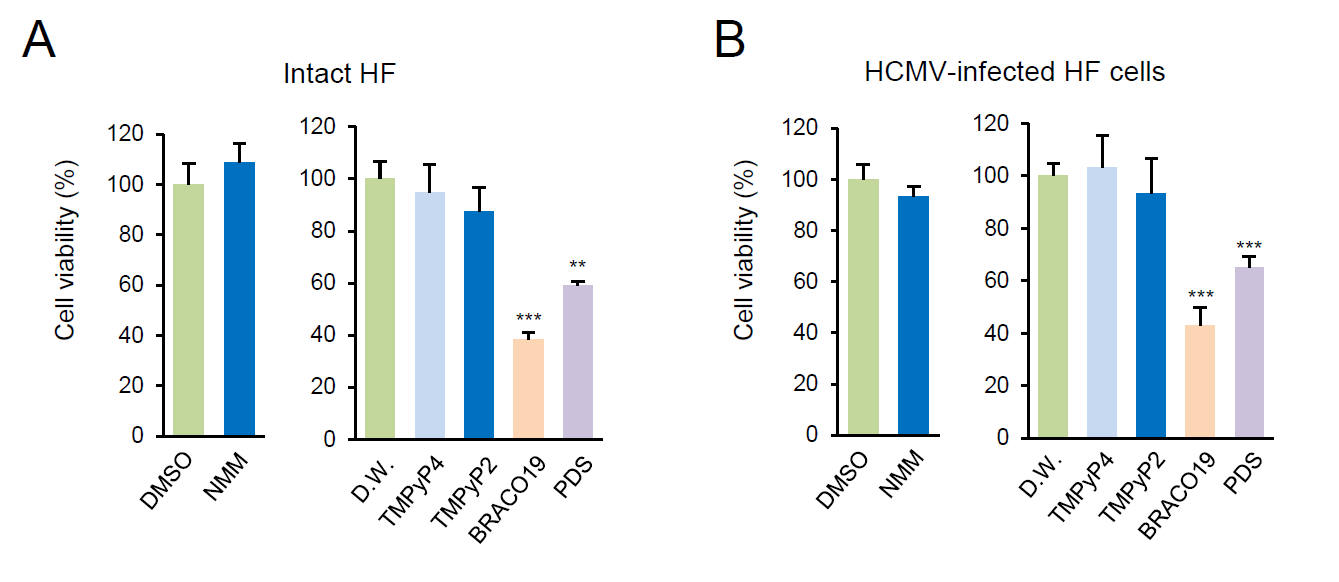

Supplement: S1 Fig — Comparison of cell viability of human fibroblast (HF) cells in the presence of various G4 ligands upon HCMV infection. Intact HF cells (A) or cells infected with HCMV(Towne) at an MOI of 1 (B) were treated with DW, DMSO, or G4-binding ligands [NMM (10 μM), TMPyP4 (10 μM), TMPyP2 (10 μM), BRACO19 (5 μM), or pyridostatin (PDS) (10 μM] as indicated for 72 h. Cell viability was measured using 3-(4,5-dimethylthiazol-2-yl)-2,5-diphenyltetrazolium bromide (MTT) assays. The results shown are averages of triplicates with error bars. Statistical significance of samples (relative to D.W. controls) was determined using the t-test, and p-values < 0.05 (*), 0.01 (**), and 0.001 (***) are indicated. (TIF) [file ppat.1007334.s002.tif]

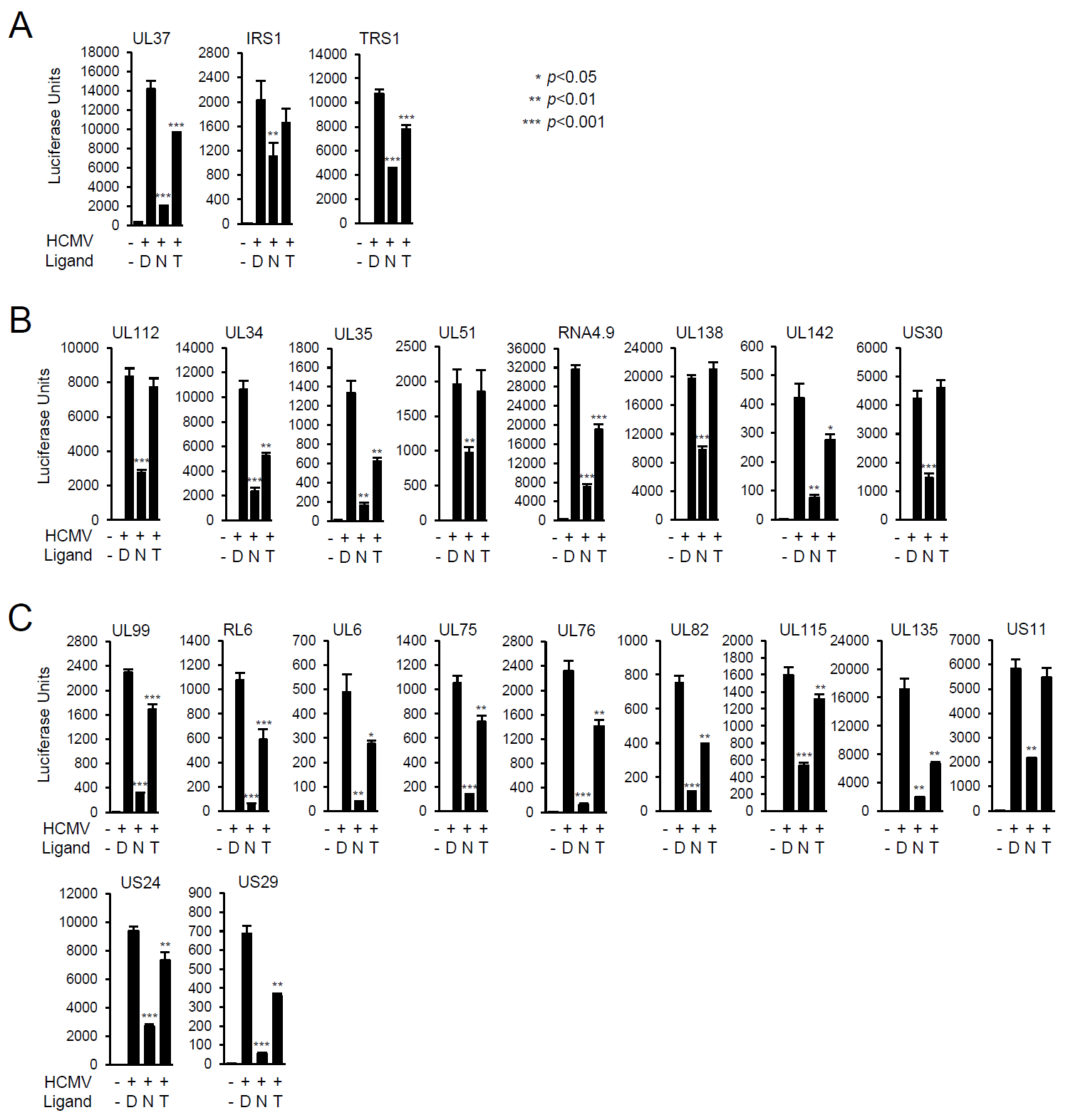

Supplement: S2 Fig — Comparison of raw luminescence values from luciferase assays with specific gene regulatory region-containing reporter constructs in the presence of G4 ligands. The luciferase units obtained from luciferase reporter assays in Fig 5B are shown here. Statistical significance of samples (relative to DMSO controls) was determined using the t-test, and p-values < 0.05 (*), 0.01 (**), and 0.001 (***) are indicated. (A) Immediate-early genes. (B) Early genes. (C) Late genes. D, DMSO; N, 5 μM of NMM; T, 5 μM of TMPyP2. (TIF) [file ppat.1007334.s003.tif]

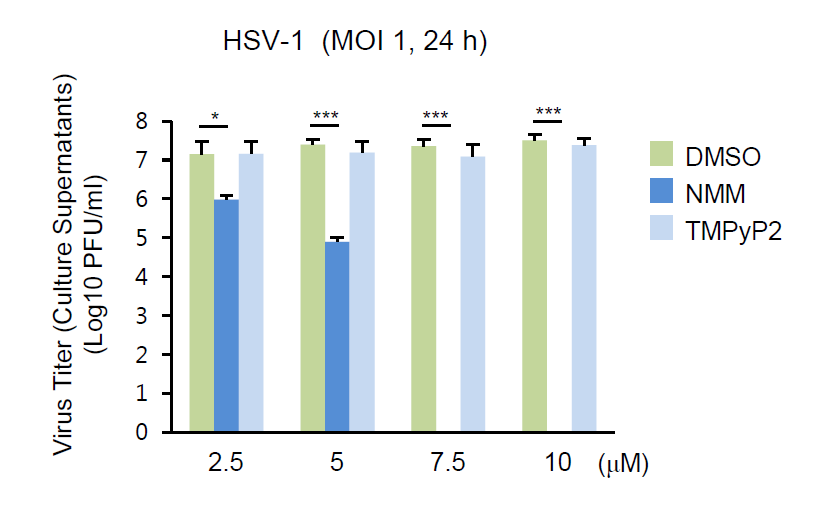

Supplement: S3 Fig — HF cells were infected with HSV-1 at an MOI of 1 and treated with DMSO (as a control) or increasing concentrations of NMM or TMPyP2. At 24 h after infection, the culture supernatants were collected at 24 h and virus titers were determined using plaque assays in Vero cells. (TIF) [file ppat.1007334.s004.tif]

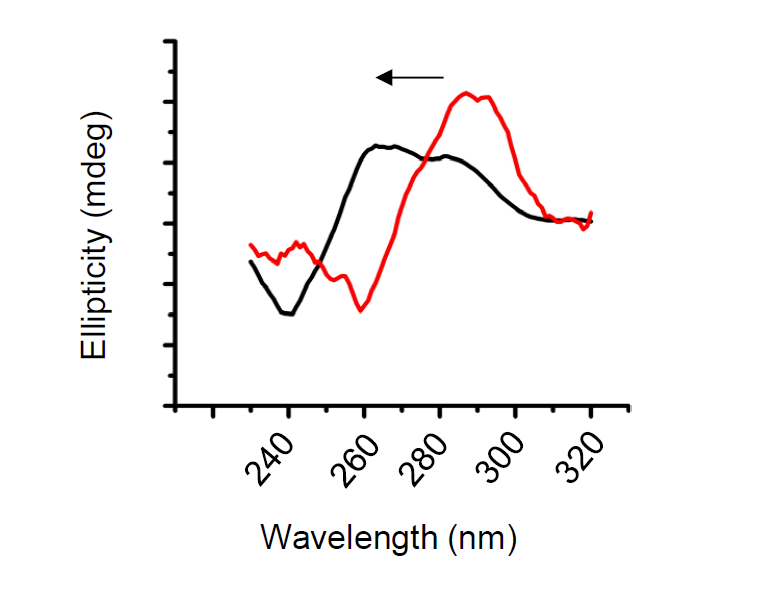

Supplement: S4 Fig — Fifteen μM DNA GQ18 oligonucleotides were annealed in the presence of 10 mM Tris-HCl [pH 7.5] and 100 mM NaCl or KCl buffer. The CD spectrum in NaCl buffer (black) is compared with that obtained in KCl buffer (red). (TIF) [file ppat.1007334.s005.tif]

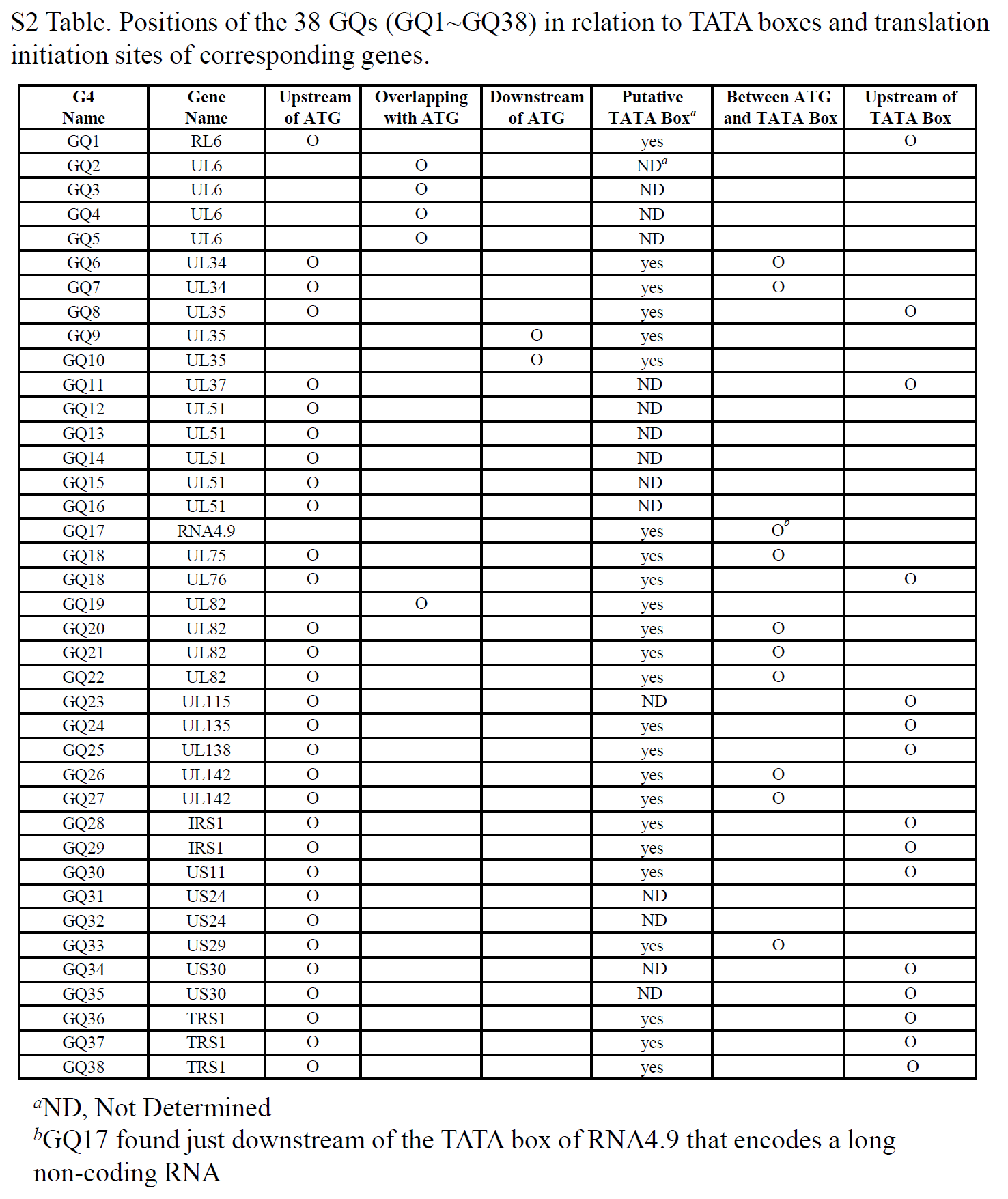

Supplement: S2 Table — (TIF) [file ppat.1007334.s007.tif]

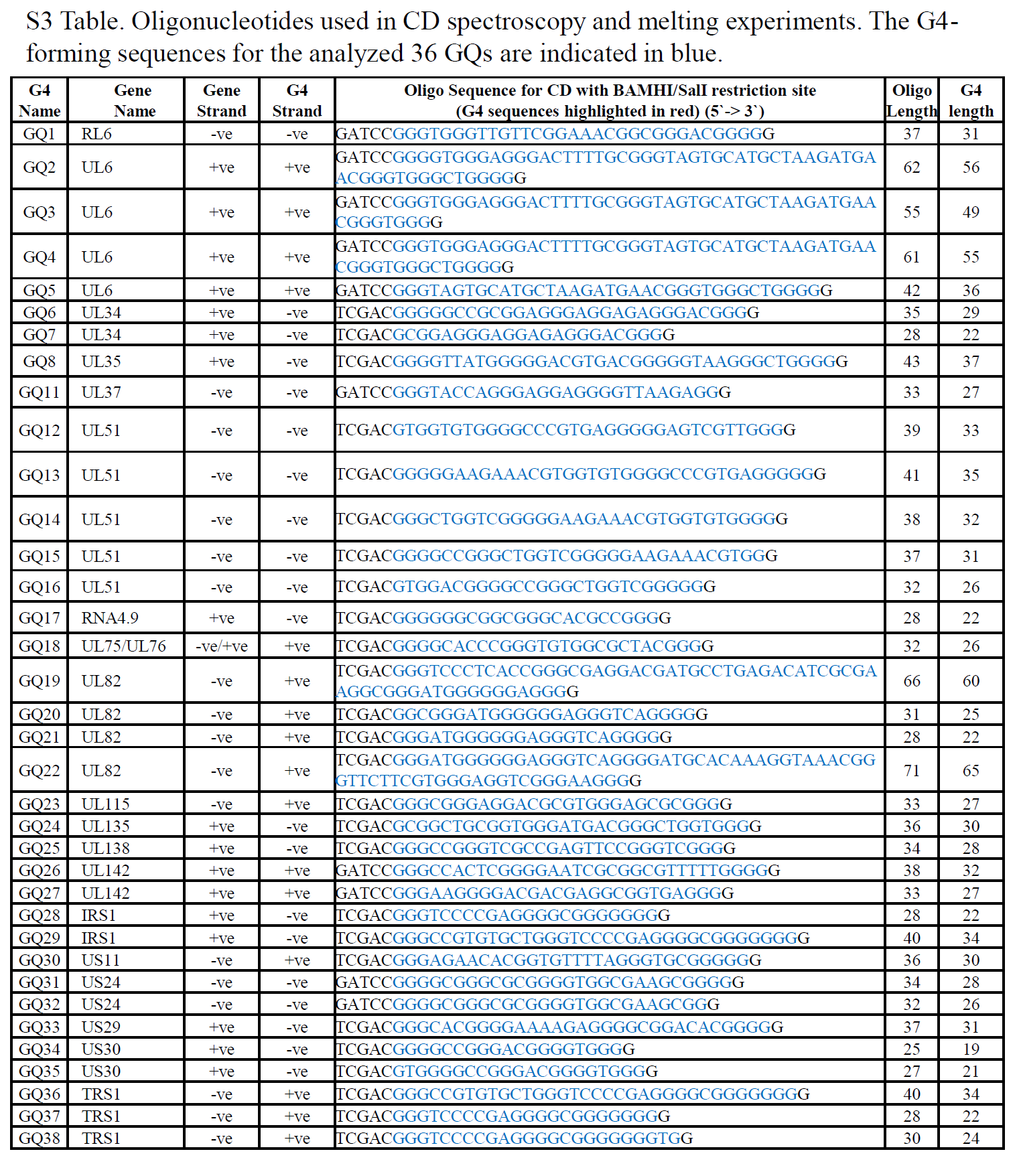

Supplement: S3 Table — The G4-forming sequences for the analyzed 36 GQs are indicated in blue. (TIF) [file ppat.1007334.s008.tif]

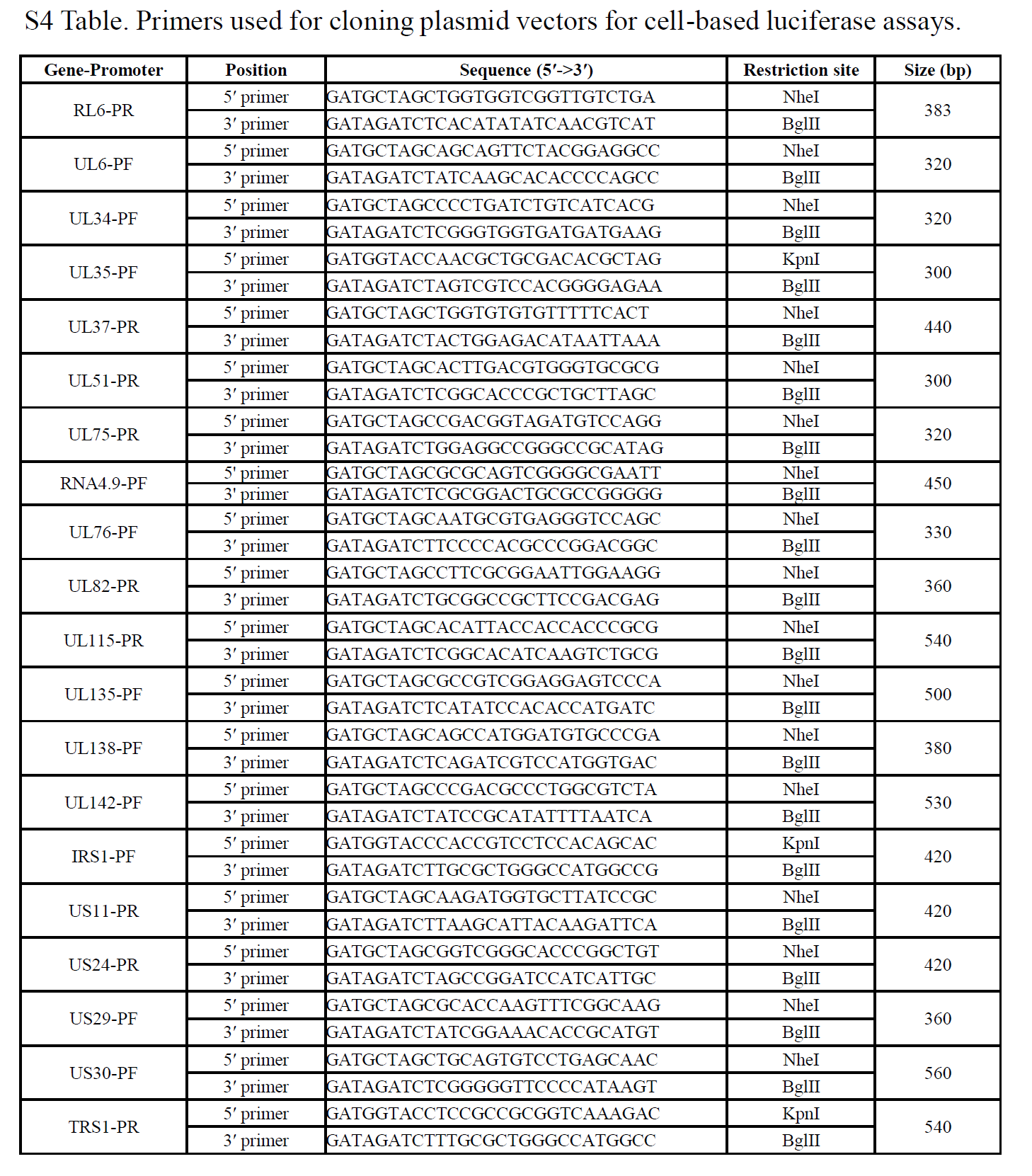

Supplement: S4 Table — (TIF) [file ppat.1007334.s009.tif]

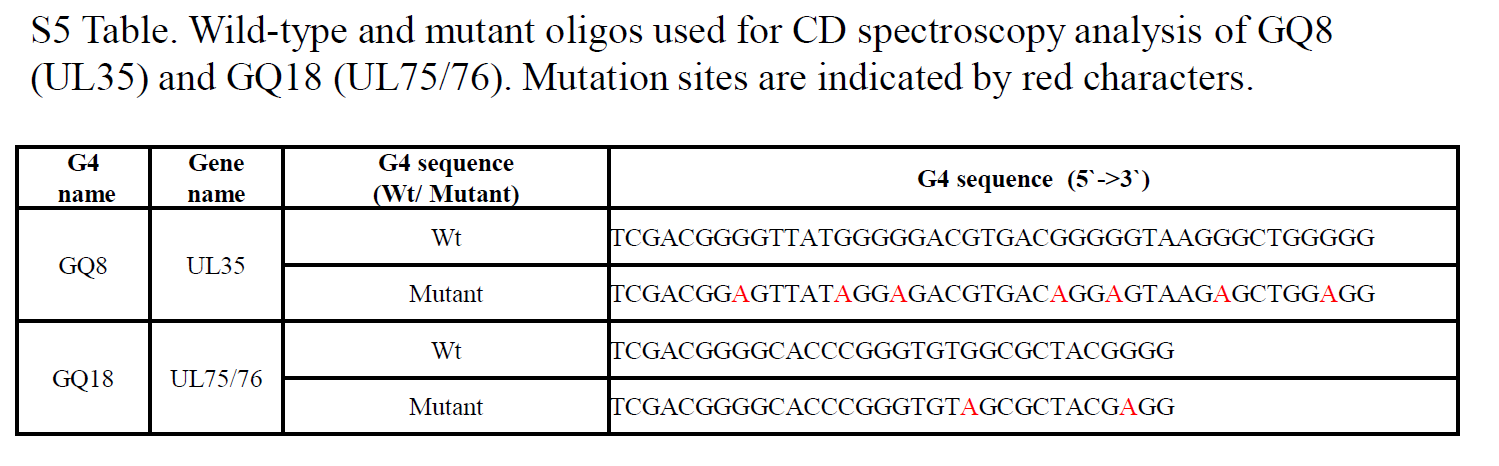

Supplement: S5 Table — Mutation sites are indicated by red characters. (TIF) [file ppat.1007334.s010.tif]
