# Supplementary material for: Genome-wide analysis of regulatory G-quadruplexes affecting gene expression in human cytomegalovirus
Source: PLoS Pathog. 2018 Sep 28;14(9):e1007334. doi: 10.1371/journal.ppat.1007334 (PMC6179306; doi:10.1371/journal.ppat.1007334)
Supplement: S1 Table — (PDF) [file ppat.1007334.s006.pdf]

S1 Table. Sequences, types, and G4 strand positions of the putative 38 GQs (GQ1~GQ38) identified in the putative regulator regions of HCMV genome (-500 and +100 with respect to the translation initiation sites).

**G4 Name**	**G4 sequence (5`-> 3`)**	**G4 start**	**G4 end**	**Type[a]**	**Gene Name (category)**	**Gene strand**	**G4 strand**	**Gene growth requirement**
GQ1	GGGTGGGTTGTTCGGAAACGGCGGGACGGGG	6548	6578	Type-II	RL6 (L)	-ve	-ve	Non-essential
GQ2	GGGGTGGGAGGGACTTTTGCGGGTAGTGCATGCTAAGATGAACGGGTGGGCTGGGG	14595	14650	Type-II	UL6 (L)	+ve	+ve	Non-essential
GQ3	GGGTGGGAGGGACTTTTGCGGGTAGTGCATGCTAAGATGAACGGGTGGG	14596	14644	Type-II		+ve	+ve	
GQ4	GGGTGGGAGGGACTTTTGCGGGTAGTGCATGCTAAGATGAACGGGTGGGCTGGGG	14596	14650	Type-II		+ve	+ve	
GQ5	GGGTAGTGCATGCTAAGATGAACGGGTGGGCTGGGG	14615	14650	Type-II		+ve	+ve	
GQ6	GGGGGCCGCGGAGGGAGGAGAGGGACGGG	44731	44759	Type-I	UL34 (E)	+ve	-ve	Essential
GQ7	GCGGAGGGAGGAGAGGGACGGG	44731	44752	Type-III		+ve	-ve	
GQ8	GGGGTTATGGGGGACGTGACGGGGGTAAGGGCTGGGG	45992	46028	Type-I	UL35 (E)	+ve	-ve	Augmenting
GQ9	GGGGAGAACGGGCAGTGGCGGGCCCGATGGGG	46139	46170	Type-I		+ve	-ve	
GQ10	GGGCAGTGGCGGGCCCGATGGGG	46139	46161	Type-III		+ve	-ve	
GQ11	GGGTACCAGGGAGGAGGGGTTAAGAGG	53120	53146	Type-III	UL37 (IE)	-ve	-ve	Essential
GQ12	GTGGTGTGGGGCCCGTGAGGGGGAGTCGTTGGG	74797	74829	Type-III	UL51 (E)/ UL52 (E)	-ve	-ve	Essential/ Essential
GQ13	GGGGGAAGAAACGTGGTGTGGGGCCCGTGAGGGGG	74807	74841	Type-III		-ve	-ve	
GQ14	GGGCTGGTCGGGGGAAGAAACGTGGTGTGGGG	74819	74850	Type-III		-ve	-ve	
GQ15	GGGGCCGGGCTGGTCGGGGGAAGAAACGTGG	74826	74856	Type-III		-ve	-ve	
GQ16	GTGGACGGGGCCGGGCTGGTCGGGGG	74837	74862	Type-III		-ve	-ve	
GQ17	GGGGGGCGGCGGGCACGCCGGG	93956	93977	Type-III	RNA4.9 (E)	+ve	-ve	Essential

S1 Table. Sequences, types, and G4 strand positions of the putative 38 G4s (GQ1~GQ38) identified in the putative regulator regions of HCMV genome (-500 and +100 with respect to the translation initiation sites) (continued).

G4 Name	G4 sequence(5`-> 3`)	G4 start	G4 end	Type[a]	Gene Name (category)	Gene strand	G4 strand	Gene growth requirement
GQ18	GGGGCACCCGGGTGTGGCGCTACGGG	111144	111169	Type-III	UL75 (L) /UL76 (L)	-ve	+ve	Essential/ Essential or Augmenting
GQ19	GGGTCCCTCACCGGGCGAGGACGATGCCTGAGACATCGCGAAGGCGGGATGGGGGGAGGG	120078	120137	Type-II	UL82 (L)	-ve	+ve	Augmenting
GQ20	GGCGGGATGGGGGGAGGGTCAGGGG	120120	120144	Type-III		-ve	+ve	
GQ21	GGGATGGGGGGAGGGTCAGGGG	120123	120144	Type-I		-ve	+ve	
GQ22	GGGATGGGGGGAGGGTCAGGGGATGCACAAAGGTAAACGGGTTCTTCGTGGGAGGTCGGGAAGGG	120123	120187	Type-II		-ve	+ve	
GQ23	GGGCGGGAGGACGCGTGGGAGCGCGGG	165952	165978	Type-III	UL115 (L)	-ve	+ve	Essential
GQ24	GCGGCTGCGGTGGGATGACGGGCTGGTGGG	176525	176554	Type-III	UL 135 (L)	+ve	-ve	
GQ25	GGGCCGGGTCGCCGAGTTCCGGGTCGGG	178404	178431	Type-II	UL138 (E)	+ve	-ve	
GQ26	GGGCCACTCGGGGAATCGCGGCGTTTTTGGGG	182177	182208	Type-III	UL142 (E)	+ve	+ve	
GQ27	GGGAAGGGGACGACGAGGCGGTGAGGG	182286	182312	Type-III		+ve	+ve	
GQ28	GGGTCCCCGAGGGGCGGGGGGG	195182	195203	Type-I	IRS1 (IE)	+ve	-ve	Non-essential
GQ29	GGGCCGTGTGCTGGGTCCCCGAGGGGCGGGGGGG	195182	195215	Type-II		+ve	-ve	
GQ30	GGGAGAACACGGTGTTTTAGGGTGCGGGGG	206242	206271	Type-III	US11 (L)	-ve	+ve	Non-essential
GQ31	GGGGCGGGCGCGGGGTGGCGAAGCGGGG	221153	221180	Type-III	US24 (L)	-ve	-ve	Augmenting
GQ32	GGGGCGGGCGCGGGGTGGCGAAGCGG	221155	221180	Type-III		-ve	-ve	
GQ33	GGGCACGGGGAAAAGAGGGGCGGACACGGGG	226086	226116	Type-I	US29 (L)	+ve	-ve	Non-essential
GQ34	GGGGCCGGGACGGGGTGGG	226935	226953	Type-I	US30 (E)	+ve	-ve	Non-essential
GQ35	GTGGGGCCGGGACGGGGTGGG	226935	226955	Type-III		+ve	-ve	
GQ36	GGGCCGTGTGCTGGGTCCCCGAGGGGCGGGGGGG	234657	234690	Type-II	TRS1 (IE)	-ve	+ve	Augmenting
GQ37	GGGTCCCCGAGGGGCGGGGGGG	234669	234690	Type-I		-ve	+ve	
GQ38	GGGTCCCCGAGGGGCGGGGGGGTG	234669	234692	Type-III		-ve	+ve	

[a]Type-I (conventional); type-II (long-loop), and type-III (bulged)
